# Keeping Nitrogen Use in China within the Planetary Boundary Using a Spatially Explicit Approach

**DOI:** 10.1021/acs.est.4c00908

**Published:** 2024-05-23

**Authors:** Xi Chen, Maryna Strokal, Michelle T. H. van Vliet, Ling Liu, Zhaohai Bai, Lin Ma, Carolien Kroeze

**Affiliations:** † Key Laboratory of Agricultural Water Resources, Hebei Key Laboratory of Soil Ecology, Center for Agricultural Resources Research, Institute of Genetics and Developmental Biology, Chinese Academy of Sciences, 286 Huaizhong Road, Shijiazhuang 050021, China; ‡ Water Systems and Global Change Group, Wageningen University & Research, Droevendaalsesteeg 4, 6708 PB Wageningen, The Netherlands; § Institue of Urban Environment, Chinese Academy of Sciences, 1799 Jimei Road, Xiamen 361021, China; ∥ Department of Physical Geography, Utrecht University, P.O. Box 80.115, 3508 TC Utrecht, The Netherlands; ⊥ State Key Laboratory of Pollution Control and Resource Reuse, School of the Environment, Nanjing University, Nanjing, Jiangsu 210023, China; # Environmental Systems Analysis Group, Wageningen University & Research, Droevendaalsesteeg 4, 6708 PB Wageningen, The Netherlands

**Keywords:** nitrogen, planetary boundary, spatially explicit boundary, water quality, food production

## Abstract

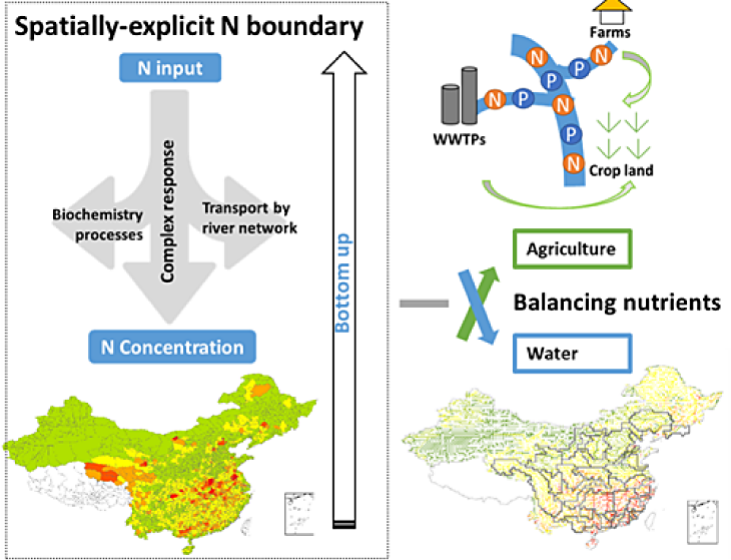

Nitrogen (N) supports food production, but its excess causes water pollution. We lack an understanding of the boundary of N for water quality while considering complex relationships between N inputs and in-stream N concentrations. Our knowledge is limited to regional reduction targets to secure food production. Here, we aim to derive a spatially explicit boundary of N inputs to rivers for surface water quality using a bottom-up approach and to explore ways to meet the derived N boundary while considering the associated impacts on both surface water quality and food production in China. We modified a multiscale nutrient modeling system simulating around 6.5 Tg of N inputs to rivers that are allowed for whole of China in 2012. Maximum allowed N inputs to rivers are higher for intensive food production regions and lower for highly urbanized regions. When fertilizer and manure use is reduced, 45–76% of the streams could meet the N water quality threshold under different scenarios. A comparison of “water quality first” and “food production first” scenarios indicates that trade-offs between water quality and food production exist in 2–8% of the streams, which may put 7–28% of crop production at stake. Our insights could support region-specific policies for improving water quality.

## Abstract 2

A new spatially explicit boundary of N for China is derived to provide insights into a posing question: how to balance nitrogen use in agriculture and nitrogen pollution in water systems?

## 1 Introduction

Human pressures on the earth system have reached an unprecedented level, exceeding the safe operating space for humanity. This especially holds for the biogeochemical flow of nitrogen (N).^1^ The planetary boundary of N (PB) was proposed based on criteria such as an N surface water quality threshold, NH_3_ (ammonia) emission, and N requirements for crop production.^1−3^ The surface water quality threshold of N for preventing widespread eutrophication of freshwater and deterioration of water quality is found as a major stringent controlled criterion in defining the PBs.^3,4^ Keeping N use within the PB is vital and urgent because the current status of using N exceeds at least twice the boundary level globally. There is a distinct need for clear PB targets at more detailed spatial levels (i.e., region, and subregion level), at which the agricultural production and N management policies are operated.^5,6^

In general, two categories of PB are distinguished, depending on their dominant functioning of the human–environmental system.^5^ The first category needs to be addressed from a global view due to its overall global impact on the earth system regardless of where on earth the emissions are generated. This category includes climate change, ocean acidification, and atmospheric ozone depletion. The second category needs to be addressed on a regional scale, as its human perturbations impact local environmental systems and thus influence global earth systems. For these boundaries, the national share in the planetary “safe operating space” is not a simple matter of allocation from global budgets. This is because the local conditions vary strongly and play a crucial role in determining the level of sustainable use and tolerable N emissions. Altered biogeochemical flows of N belong to this category.

Several studies assessed the planetary boundary of N for water quality at regional scales.^2−4,7^ However, current studies still rely on either aggregated national values or a simplified approach to derive regional boundaries. For example, Yu et al.^2^ proposed nationally aggregated estimates of N inputs to rivers as a safe N boundary for China. However, the complex response of the N concentration to N inputs to rivers is omitted. It makes it difficult to conclude that the surface water quality of N for the whole nation or province is safe as long as the total N inputs are within the boundary. In reality, the N concentrations for certain regions may not be well below the threshold, particularly for highly urbanized and agricultural regions.^8^ This is because keeping N concentrations below the threshold for each region requires accounting for the combined effects of nutrient inputs to rivers, the biochemistry of nutrients in rivers (retentions), and the transport of nutrients by the river network. Similarly, Schulte-Uebbing et al.^3^ did not quantify the regional N boundary based on N concentrations in surface waters. Instead, the study considered the N inputs dividing the runoff as the proxy for in-stream N concentrations using a simplified approach. Hence, we argue that a country’s share in the planetary boundary of N should be assessed and recommended based on a spatially explicit approach that explicitly include complex relationships between N inputs and in-stream N concentrations.

China has dramatically increased its N use in the past few decades.^9−11^ Nutrients dominate Chinese surface water pollution.^12^ Spatially explicit estimates of the N boundary (e.g., county or sub-basin level) are more meaningful to manage N use to restore China’s surface water quality. Management strategies are proposed such as reducing the overapplied synthetic N fertilizer and increasing the recycling rate of N in manure.^13^ The regional N boundary could help to explore to what extent these management strategies should be applied and estimate their associated spatially explicit impacts on surface water quality. However, N is not only contributing to water pollution but is also an indispensable element of modern agriculture.^14,15^ Crop production has been the priority of China’s central government to ensure food security.^16,17^ The management strategies to reduce N use to improve water quality may compromise food production.^18,19^ Therefore, the improved understanding of the potential trade-offs between surface water quality and food production from reducing current N use through the food chain of China is also essential.

Therefore, the objective of this study is to (1) derive a spatially explicit boundary of N for surface water quality and (2) explore management strategies to meet the derived N boundary while considering the associated impacts on both surface water quality and food production. To this end, we developed and applied a bottom-up approach and modified the MARINA 3.0 (Model to Assess River Inputs of Nutrients to seAs) model^8,20^ together with the NUFER (NUtrient flows in Food chains, Environment, and Resources use) county model^11,21^ and VIC (Variable Infiltration Capacity) hydrological and RBM (River Basin Model) water temperaure^22−24^ to incorporate this approach to derive a spatially explicit regional N boundary for China.

## 2 Methodology

We developed a new approach to define and meet the spatially explicit boundary of N inputs to rivers in China following the surface water quality threshold. As opposed to a more “top-down” approach to allocate China’s share in PB,^5^ we develop here a “bottom-up” approach. In our bottom-up approach, we define China’s share in the PB for N as maximum allowable N inputs to rivers based on surface water quality of N. This requires the complex response of N in-stream concentrations (details in Section S1.2) to N inputs to rivers (details in Section S1.1). The derived spatially explicit N boundary is then aggregated from streamlines (0.5° river routing network) to county, province, and nation (details in Section S1.4) by the multiscale MARINA 3.0 model of Chen et al.^20^ Agricultural management strategies (i.e., reduction of fertilizer and manure) are applied to keep N use within the newly defined spatially explicit boundary while taking into account the N requirement of crop production. In this way, we evaluate the impacts of the management options for keeping N use within the derived boundary on both surface water quality and food production. In the following sections, we describe our bottom-up approach, including the descriptions of the model and developed scenarios.

### 2.1 Model Description and Bottom-Up Approach

#### 2.1.1 Model Description

The spatially explicit multiscale surface water quality model for nutrients, named MARINA 3.0 (Model to Assess River Inputs of Nutrients to seAs, also referred as MARINA-Nutrients, China-3.0),^8,20^ has been modified to quantify the regional boundary of N inputs to rivers for keeping the surface water quality within the planetary boundary. The model has been developed for in-streamwater quality assessment, including different nutrient forms and sources. The model first quantifies annual flows of nutrients from land to streams (including, e.g., fertilizer and manure applied), followed by retentions of nutrients and their transport by the river network.^8^ Our model framework (Figure S1) is developed based on three existing modeling approaches namely MARINA 1.0,^25^ NUFER county,^11,21^ VIC hydrological, and RBM water temperature,^22−24,26^ models. **For details of MARINA 3.0 (including the improvements compared to older versions), we refer to**Sections S1.1 and S1.2. Below, we briefly summarize how the model has been modified for deriving the spatially explicit N boundary and keeping surface water quality within the derived N boundary under different bottom-up scenarios for the year 2012.

#### 2.1.2 Bottom-Up Approach

Studies such as Steffen et al.^1^ defined a planetary boundary (PB) for N as the amount of human intended N fixation applied to soil, which will prevent widespread eutrophication of freshwater and deterioration of water quality.^1,4^ Human-intended N fixation includes biological N fixation (BNF) and mineral N fertilizer use. We include more N sources that affect surface water quality than human-intended N in our approach, including (see details in Section S1.1): 1) diffuse sources; 2) human waste from wastewater treatment plants (WWTPs); 3) point source discharge of manure; and 4) human waste (unconnected sewage).

We use the modeled dissolved inorganic nitrogen (DIN) concentration (details in Section S1.2) in the bottom-up approach to set the regional N boundary for Chinese streams. The threshold of 1 mg/L is selected for DIN based on the reviews of the ecological and toxicological impacts of inorganic N pollution, an overview of maximum allowable DIN concentrations and the national standard for N concentrations in surface waters.^2,4,27−29^ We addressed the associated uncertainties in the following discussion section.

**Figure 1:**
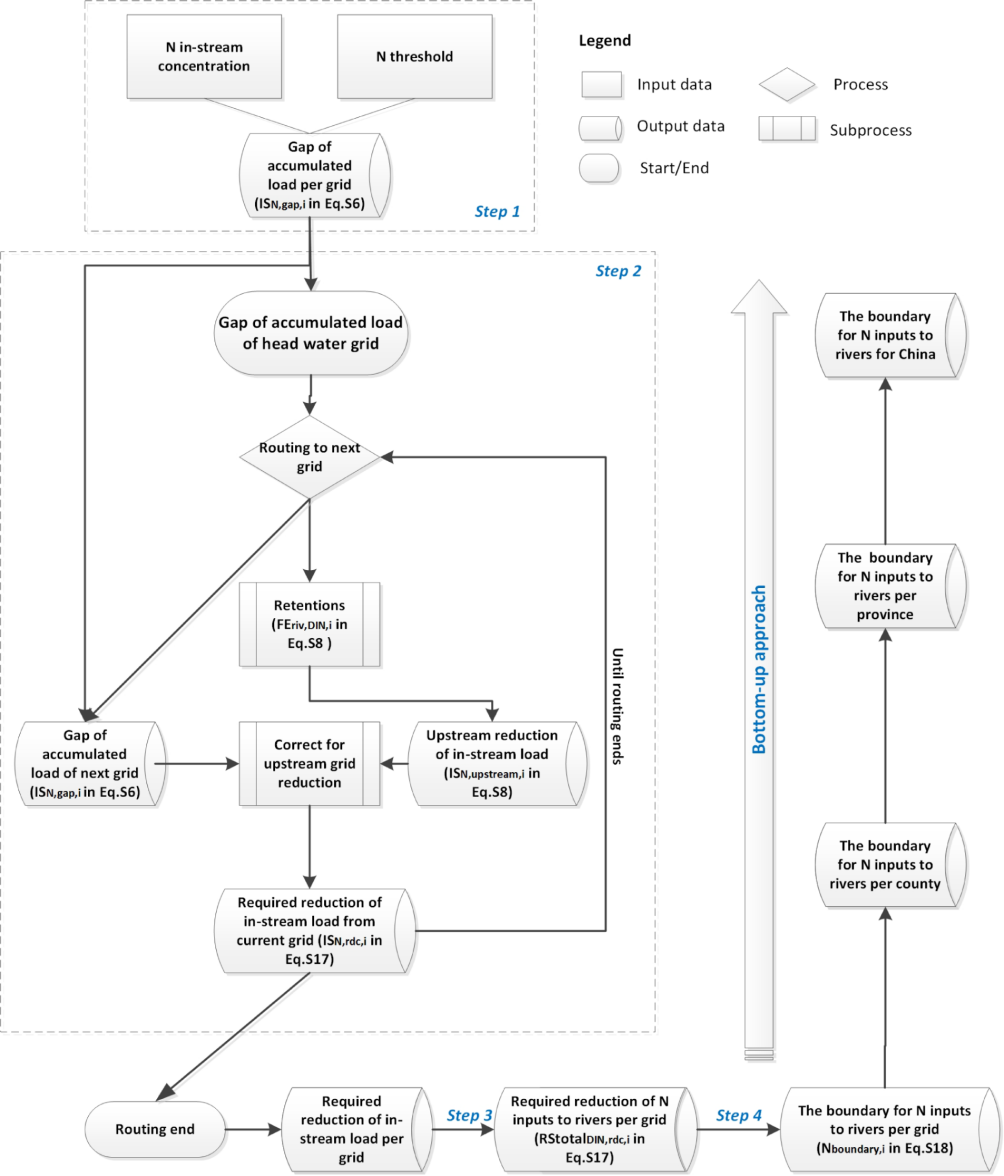
A graphical scheme of the bottom-up approach for calculating the regional nitrogen (N) boundary for inputs of N to rivers based on the surface water quality threshold. The descriptions of the parameters and variables between brackets refer to explanations of the associated equations in the Supporting Information. The **first step** is to quantify the gap of accumulated load (in-stream load, kton/year) based on the gap of in-stream N concentrations and N threshold by multiplying it with the water discharge for the grid cell. The **second step** is routing the calculated gap of accumulated N load along the river network to correct the upstream-to-downstream influence and thus quantify the actual required reduction of in-stream load (kton/year) from local grid cells. The **third step** is to quantify the required reduction of N inputs to rivers (kton/year) based on the required reduction of in-stream N load. The **final step** is to quantify the regional N boundary (kton/year) by using current N inputs to rivers minus the required reduction of N inputs to rivers. The associated quantifications of step 1 to step 4 are described in detail in Section S1.4.

China’s share in the PB of N for surface water quality is derived based on the gap between the current surface water DIN concentration and the critical threshold of 1 mg/L. This requires quantifying the gap between the local water quality level and the targeted threshold while taking into account the complex response of N in-stream concentrations to N inputs to rivers. Figure 1 illustrates the general steps of the bottom-up approach to quantifying the regional boundary of N for surface water quality.

First, we quantify the gap of accumulated load (in-stream load), which is the gap between the current in-stream N concentration per grid cell and the critical threshold per grid cell by multiplying it with the discharge per 0.5°grid cell (**step 1**). The gap of the accumulated load is not yet equal to the actually required reduction of in-stream N load for meeting the surface water quality threshold due to the upstream-to-downstream impacts. Next, the gap of the accumulated load is routed along with the river network with retentions to correct the impact of upstream grid cell reduction on downstream grid cells (**step 2**). In this way, we quantify the required reduction of the in-stream load from local grid cells. Third, we quantify the required reduction of N inputs to rivers of each grid cell by dividing the actual required reduction of in-stream load per grid cell by the fraction of river export of that grid cell, which incorporates the retention processes in river networks (**step 3**). Finally, the current N inputs to rivers per grid cell minus the actual required reduction of N inputs to rivers per grid cell is the regional N boundary based on the surface water threshold for China (**step 4**). **The associated quantifications of step 1 to step 4 are described in detail in**Section S1.4.

#### 2.1.3 Keeping Nitrogen Use in Agriculture within the Regional Boundary

We consider agricultural management to keep N use within the derived regional boundary, i.e., fulfilling the required reduction for each region (e.g., grids and counties). We identify the targeted reduction sources from agriculture, which include synthetic fertilizer applied on agricultural land and manure applied on agricultural land. We assume that other N sources do not change with reduction options. These sources are biological N fixation, N deposition, human waste applied on land, and human waste emitted from WWTPs

Two rules are considered when reducing agricultural N use:

1. The use of synthetic fertilizers and animal manure should first ensure crop production (details in S1.5) and then be reduced to meet the water quality threshold.
2. First ensure water quality meets the threshold via reducing agricultural N inputs to land (synthetic fertilizers and animal manure); crop requirements could be compromised.

**We refer to details of how we quantify the required reduction of N use from land based on above two rules in**Section S1.5.

### 2.2 Scenario Description

**Figure 2:**
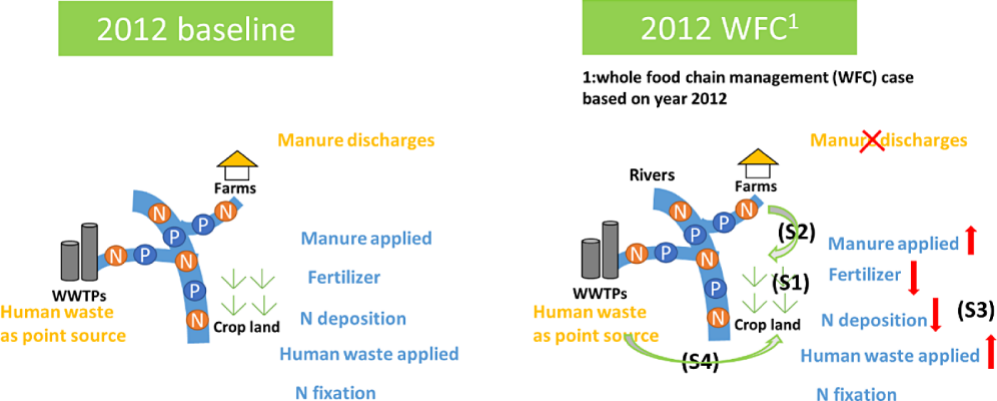
Schematic illustration of the two reference scenarios including the Baseline for the year 2012 and Whole Food Chain (WFC) management scenarios^30^ based on the year 2012. WWTPs refer to wastewater treatment plants. (S1) refers to management strategy 1, i.e., increasing soil fertility; (S2) refers to management strategy 2, i.e., abandoning discharge of manure and increasing recycling of manure; (S3) refers to improved livestock manure management with low ammonia emission; and (S4) is to include new systems to recycle human excretion and food waste. The red arrow refers to the increase or decrease of N sources in WFC compared to the Baseline scenario.

We developed eight scenarios. Two of these are reference scenarios: (1) the Baseline for the year 2012 and (2) the Whole Food Chain management (WFC) with improved nutrient management based on the reference year of 2012 (Figure 2 and Table S3). The other six scenarios are alternatives to the two reference scenarios that were developed by applying the bottom-up approach (Table S3). Below, we describe the two reference scenarios (Baseline and WFC) and the associated six alternative scenarios.

#### 2.2.1 Baseline

The Baseline scenario is the situation of 2012 (Figure 2 and Table S3). We have detailed information on >2300 counties for this year. The data and parameters were derived from the Data Center for Resources and Environmental Sciences, Chinese Academy of Sciences (RESDC), Wastewater Treatment Plant (WWTP) Database (>4,000 WWTPs), and literature reviews.^8,20^ We applied the MARINA 3.0 model for nutrients in-stream modeling using the setting as described in Chen et al.^20^ and Chen et al.^8^

#### 2.2.2 Whole Food Chain Management (WFC)

The WFC scenario is a nutrient management scenario based on the reference year 2012 (Figure 2 and Table S3). The WFC scenario is developed by Jin et al.^30^ using the NUFER county model, with the Whole Food Chain nutrient management to the reference year 2012. In the WFC, crop production is assumed to be the same as Baseline 2012. The nutrient management strategies in WFC include balanced fertilization, improved nutrient management in the crop–livestock sector, and improved nutrient management of N inputs from the recycling of food waste and human excreta to cropland. In addition, there are improved soil management, emission mitigation in livestock production, and enhanced collection, sanitation, and utilization of N in food waste and human excreta. The WFC scenario also accounts for a new system that will be built to collect human excretions, which used to be treated in a sewage treatment system, with the preservation and recycling of nutrients. For details, we refer to Jin et al.^30^ and the associated changed parameters in MARINA 3.0 compared to baseline (i.e., the assessment for Chen et al.^8^ is summarized in Table S1.

#### 2.2.3 Six Alternative Scenarios

Each reference scenario (Baseline and WFC, Figure 1) has three alternatives, resulting in six alternative scenarios (Table S3). Alternative scenarios incorporate the bottom-up perspective to manage pollution. These include:

1 Regional N boundary (regN) scenarios: Baseline-regN and WFC-regN. Here, the scenarios quantify the regional N boundary by applying the bottom-up approach.
2 Measure—“water quality first” (wq1st) scenarios: Baseline-wq1st and WFC-wq1st. Here, the scenarios are followed on the derived regional N boundary (regN) to first ensure water quality meets the threshold via reducing agricultural N inputs to land (synthetic fertilizers and animal manure). Crop requirements could be compromised.
3 Measure—“food security first” (food1st) scenarios: Baseline-food1st and WFC-food1st. Here, the use of synthetic fertilizers and animal manure should first ensure crop production and then be reduced to meet the water quality threshold.

## 3 Results and Discussion

### 3.1 China’s Share in the Planetary Boundary

**Figure 3:**
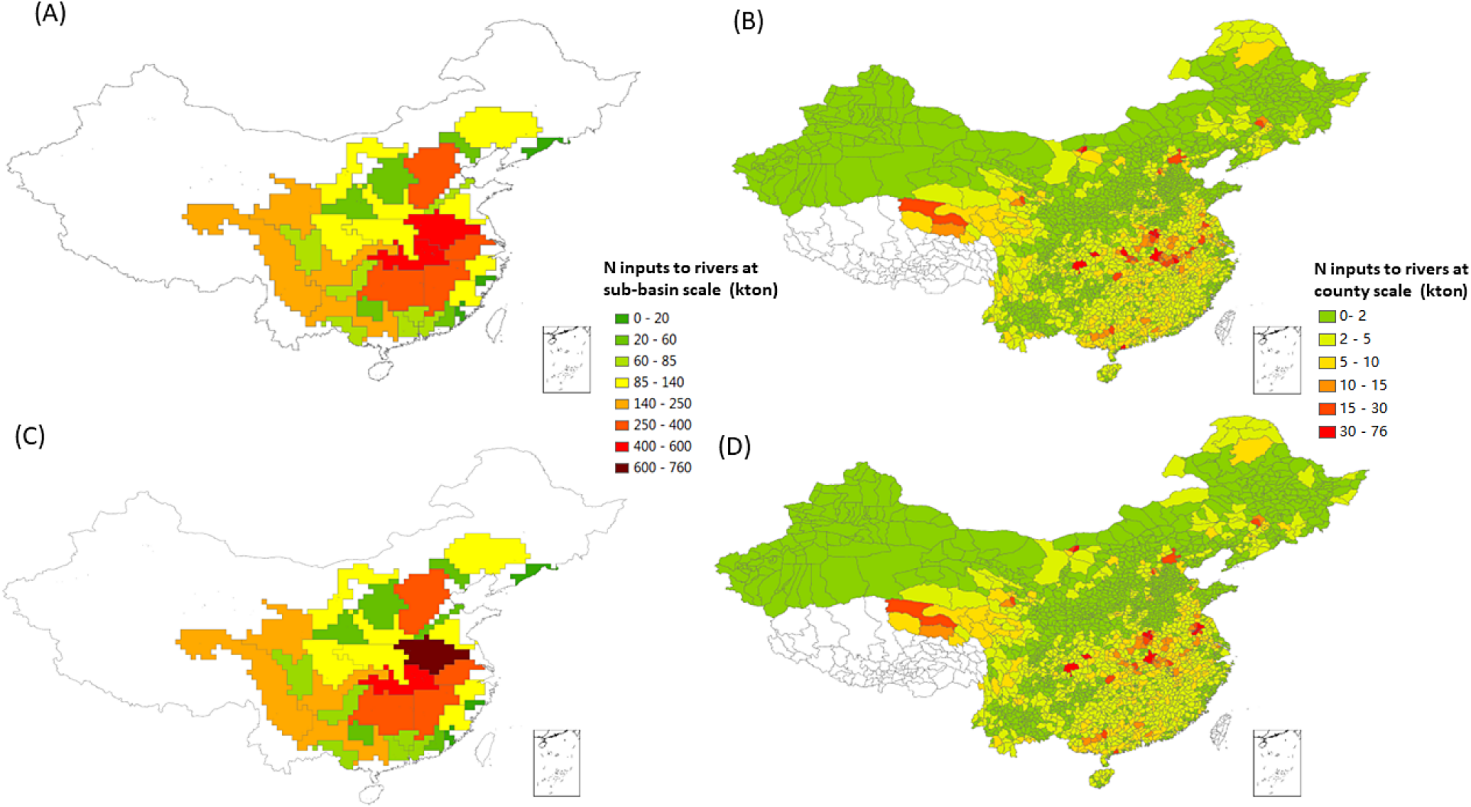
Calculated maximum levels of N inputs to rivers (kton/year) as China’s share in planetary boundaries (PB) to ensure that N concentrations do not exceed the PB threshold (1 mg/L) for the Baseline-reqN and WFC-reqN scenarios: (A). N boundaries for sub-basins for the 2012 Baseline; (B). N boundaries for counties for the 2012 Baseline; (C). N boundaries for sub-basins for the Whole Food Chain (WFC) management scenario; and (D). N Boundaries for counties for the WFC scenario. “reqN” refers to scenarios that quantify the regional N boundary by applying bottom-up approach (Section 2). The study area of subbasins is presented in Figure S3.

Our study provides a spatially explicit N boundary for China (Figure 3). The multiscale boundaries for N at sub-basin, county, and national scales are derived based on the bottom-up approach. China’s share in the planetary boundary is quantified as N inputs to rivers, ranging from 6500 to 6600 kton for the WFC and Baseline scenarios for the year 2012, respectively. The differences between the Baseline and WFC scenarios for the derived boundaries (Figure 3) and the required reduction of nutrient inputs to rivers (Figure S4) are due to the combined effects of multiple factors, such as the spatial distribution of nutrient inputs to rivers (Figure S5) and the associated retention by the river network (see extended discussions in Section S2.2). There are large regional differences in N boundaries (Figure 3). Maximum allowed N inputs to rivers are relatively high for sub-basins such as those middle and downstream of Yangtze and Hai, where intensive food production regions such as North China Plain are located. The higher N boundaries of these sub-basins, reflecting higher capacity of receiving pollution load, are resulted from the combined effects of multiple factors including the higher in-stream retentions (combined effects of water temperature, river discharge, etc.), longer transport by the river network (longer residence time to retain the nutrients), and higher river discharges (for Yangtze sub-basins). This implies that the relatively high N boundaries of these regions could potentially release pressure on the current management of food production (with the associated required reductions of N inputs to rivers ranging from 50% to 80% of these sub-basins, Figure S4). However, we calculate relatively low boundaries for urbanized coastal sub-basins such as Delta Zhujiang, Jiulong, and Han. Urban agglomerations such as Guangdong–Hong Kong–Macao (the Greater Bay Area) are located in these regions, where human activities are intensive and urbanization rates are high. This implies a delicate balance between economic development and water quality in these regions (with the associated required reductions of N inputs to rivers larger than 75%, Figure S4).

Our N boundary for China as a whole is somewhat higher than the estimate of Yu et al.^2^ of 5200 kton of N discharged to rivers. The national aggregated boundary of Yu et al.^2^ is based on the estimated provincial N inputs to rivers when the representative catchments first exceeded 1 mg/L by the mid-1980s. The lower estimates of the N boundary by Yu et al.^2^ resulted from the lower N concentrations by mid-1980s when the surface water was less polluted compared to N concentrations in year 2012 of this study. On a national scale, a 64% reduction of N inputs to rivers is needed to meet the derived N boundary based on Yu et al.,^2^ while our study presented a 65% required reduction of N inputs to rivers. The spatial distribution of the exceedance of current N inputs to rivers by derived boundaries on a provincial scale is generally in line with their estimates (see details in Section S2.2).

**Figure 4:**
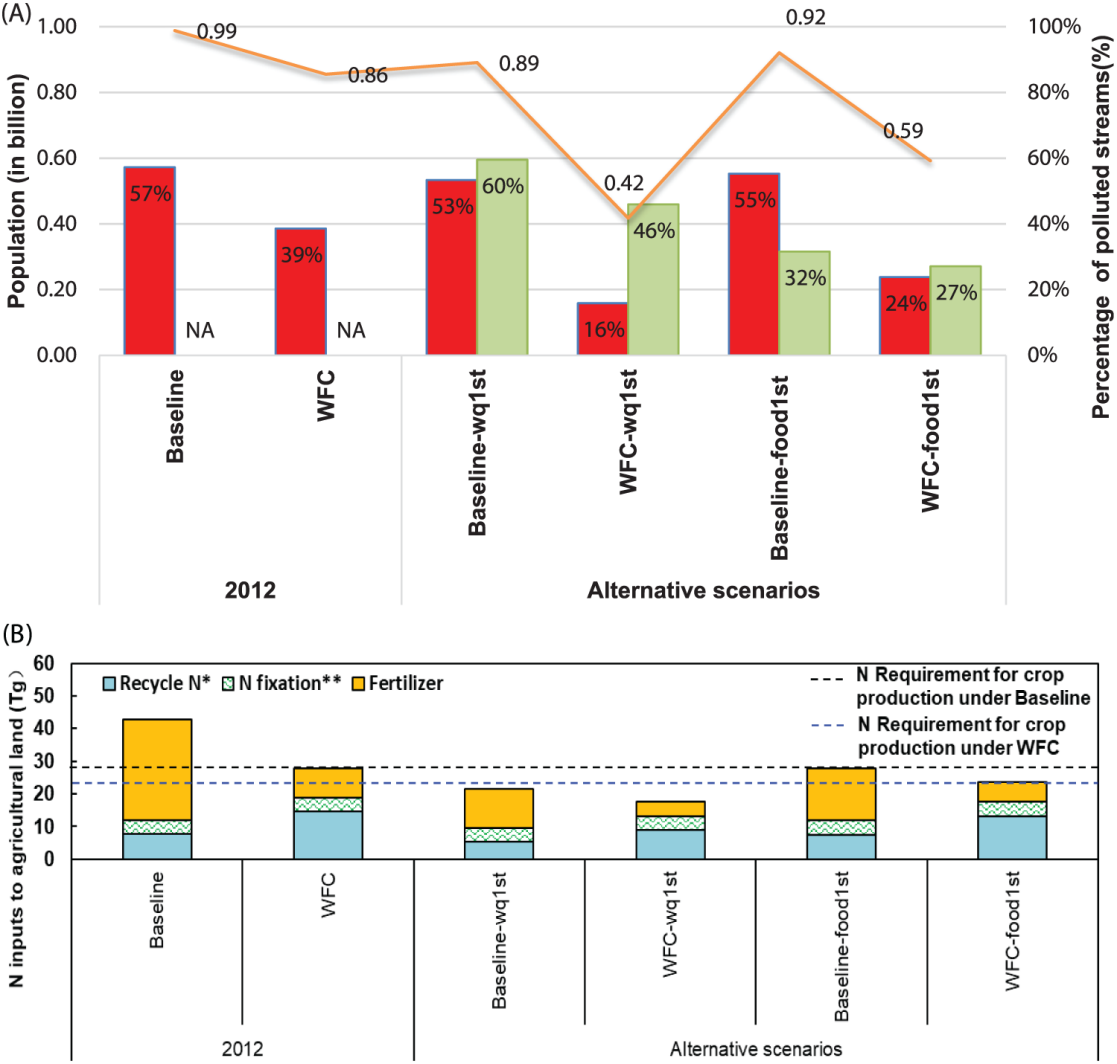
Results for different scenarios. (A) The red bars show the percentage of polluted streams (above the threshold of 1 mg/L), and the orange line shows the number of people living in these polluted watersheds (in billion). The green bars show the required reductions in synthetic fertilizers and animal manure (%) for each scenario compared to the total synthetic fertilizer and animal manure of the Baseline or Whole Food Chain (WFC) scenarios. (B) N is required to maintain current yield levels (dashed lines) and N inputs to agricultural land (bars) under the Baseline and WFC and their associated bottom-up scenarios. Note: Blue and black dashed line refers to the required N to maintain the current crop yield under Baseline and WFC scenario. *Recycled N input includes N recycled from straw, livestock manure, and human excretions, which were corrected by synthetic fertilizer value and N deposition. **N fixation refers to biological N fixation. Baseline refers to the situation of N sources in 2012; WFC refers to Whole Food Chain nutrient management and recycling. Baseline-wq1st and WFC-wq1st refer to the scenarios that first ensure water quality without considering crop needs (details in Section 2). Baseline-food1st and WFC-food1st refer to the scenarios that first ensure crop needs and then reduce the extra fertilizer and manure for meeting the water quality threshold.

Schulte-Uebbing et al.^3^ quantified the global N boundary based on thresholds for N surface water quality, N deposition, and N groundwater quality. They found that surface water quality is the most stringent criterion that requires the highest reduction of current N inputs. Schulte-Uebbing et al.^3^ presented the derived boundaries in terms of the agricultural N surpluses. Based on the threshold of 2.5 mg/L N concentration in surface water, they show that a 63% reduction in N use in agriculture is needed. Based on the threshold of 1 mg/L, we show that a 60% reduction of agricultural N use is needed (Figure 4). This implies that if we choose the same threshold (1 mg/L increase to 2.5 mg/L), the associated percentage of required reduction of agricultural N use will be lower than theirs. This confirms with their discussions regarding results in China,^3^ where it indicated that if a threshold of 1 mg/L is implemented, the model simulates that N discharge to surface water in China needs to be reduced by 71%, while our estimated 65% N inputs to rivers for China need to be reduced. This lower required reduction could result from that we consider upstream-to-downstream impacts, i.e., the reductions in upstream parts could compensate the required reduction of downstream parts, resulting in the generally lower required reductions. This phenomenon seems similar when it goes to smaller scales (Figure S4 compared to Extended data Figure 3 of Schulte-Uebbing et al.^3^). One of the limitations of our study is that we did not include other N-related criteria to derive the N boundary as for example Schulte-Uebbing et al.^3^ However, we improve their estimates by considering the different spatial characteristics of N retentions and upstream-to-downstream impacts. Additionally, we would like to highlight that restoring surface water quality is more complex and requires more actions (e.g., stepwise long-term ecological restoration that encompasses for instance environmental remediation, ecological rehabilitation, and natural recovery^31,32^) than only reduction of regional N use alone.

### 3.2 Keeping Nitrogen Use in China within the Regional Boundary

Agricultural N inputs must be managed to keep N use within the derived boundaries. Here, we focus on meeting the required reductions to keep N concentrations in rivers below 1 mg/L for each region. We focus on the most important agricultural sources of N: synthetic fertilizers and manure applied on agricultural land. We ignore possible reductions in other sources of N. We analyze the results in terms of the required reductions in N use (i.e., for fertilizer and manure), the impacts thereof on food production, and the associated impacts on surface water quality.

#### 3.2.1 Impacts on Surface Water Quality

**Figure 5:**
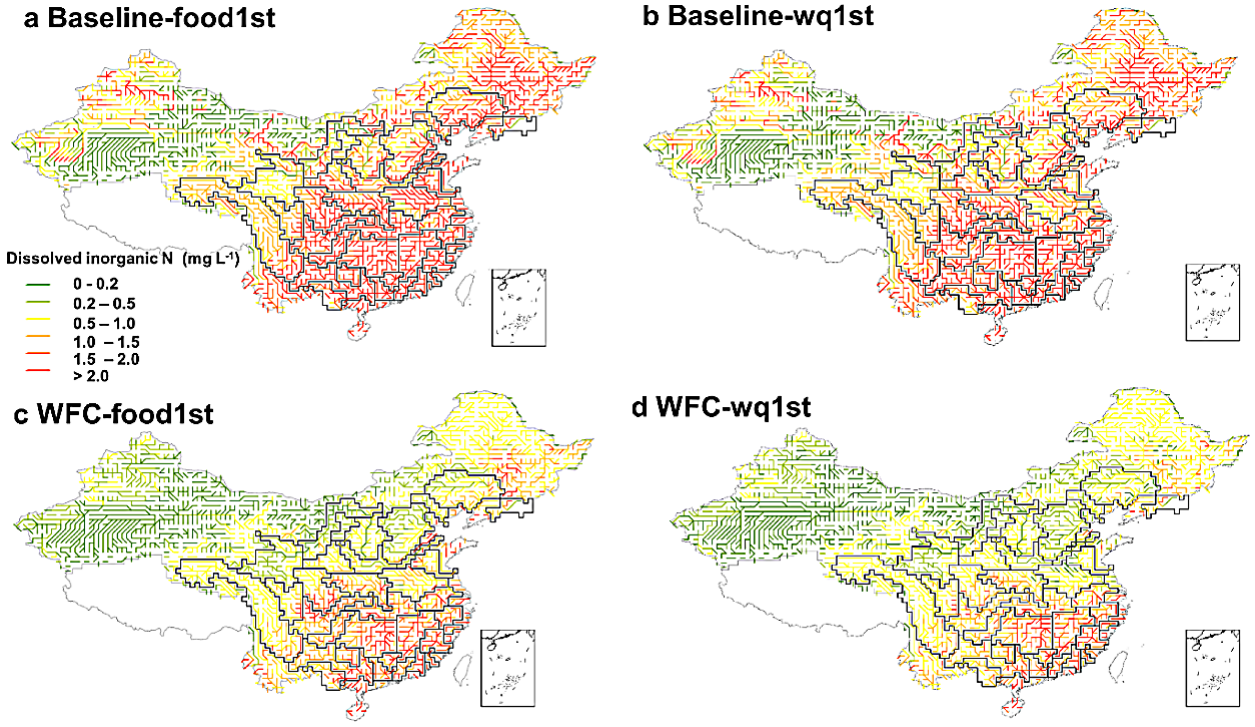
Surface water quality (in concentration of dissolved inorganic nitrogen, mg/L) under “food security first” (food1st) scenarios (a,c) and “water quality first” (wq1st) scenarios (b,d). Baseline refers to the baseline for the year 2012. WFC refers to Whole Food Chain management based on the 2012 scenario. Baseline-food1st and WFC-food1st refer to the scenarios that first ensure crop needs and then reduce the extra synthetic fertilizer and animal manure for meeting water quality threshold. Baseline-wq1st and WFC-wq1st refer to the scenarios that first ensure water quality without considering crop needs (details in Section 2). Figure S3 indicates the dessert areas, of which the interpretation of the presented concentrations should particularly consider the uncertainties of the hydrological outputs in these very dry areas.

In the baseline year 2012, 57% of streams (based on the number of streams dividing the total number of streams) are polluted, i.e., N concentration exceeds 1 mg/L (Figure 4). Reducing the overapplication of fertilizers and manure results in a 30–60% reduction in N applied compared to the Baseline 2012, depending on whether N inputs for meeting the crop requirement will be ensured (Baseline-wq1st and Baseline-food1st). These reductions in N inputs in 2012 account for around 25% of the total required reductions in N inputs to water bodies. This agrees with the study of Yu et al.^2^ that showed that improved cropland N management could reduce 25% of excess N discharges to surface water. However, these reductions restore only 2–5% of the polluted streams (Figures 4 and 5). This suggests that under the current level of nutrient use efficiencies in agriculture, restoring the surface water quality will be difficult. When considering the Whole Food Chain management of nutrient use, the percentage of polluted streams could be reduced to 39%. This is associated with higher nutrient use efficiencies and other management strategies, such as increased nutrient recycling of manure (see Figure 1). In the WFC scenario, managing fertilizer and manure for meeting the derived regional N boundary results in a 27% reduction in N inputs and a 15% reduction in polluted streams, while assuming crop production as the first priority (WFC-food1st). However, reducing fertilizer and manure to improve water quality status (i.e., without fulfilling the crop requirement, WFC-wq1st) results in a 46% reduction in fertilizer and manure and a 23% reduction in polluted streams compared to WFC (Figure 4a). This Whole Food Chain management as assumed in the WFC scenario could potentially restore current Chinese surface water quality to a large extent compared with the 2012 Baseline. The number of people living in the polluted watersheds could be halved in both WFC scenarios and only by 10% in the Baseline scenarios. The comparison between the Baseline and WFC scenarios shows that the management of fertilizers and manure alone is insufficient to solve the water pollution problem. Nutrient management through Whole Food Chain is required, including a ban on direct discharges of manure, recycling of human waste, and improved management of livestock systems, which leads to a reduction of N deposition from the agricultural sector. Our findings agree with Schulte-Uebbing et al.^3^ who concluded that feeding the world without trespassing the planetary N boundary requires not only large increases in nitrogen use efficiencies but also mitigation of nonagricultural N sources.

#### 3.2.2 Impacts on Food Production

The Baseline-food1st and WFC-food1st scenarios only reduce the agricultural N inputs under the condition of meeting crop production needs (Figure 4). In Baseline-wq1st and WFC-wq1st, the reduction of synthetic fertilizers and animal manure could achieve further 5% and 10% reduction of polluted streams compared to Baseline-food1st and WFC-food1st, respectively. However, this requires around 13–28% further reductions in the agricultural N inputs to rivers compared to that of Baseline and WFC, respectively. The comparisons between “water quality first” (wq1st) and “food security first” (food1st) elucidate potential trade-offs between crop production and water quality. The comparisons indicate that the trade-offs between water quality and food production exist in 2–8% of the streams, which put 7–28% of the crop production at stake, depending on the Baseline or WFC scenarios (Figure 5) (details in Section S2.2). The streams with trade-offs are largely located in upstream and downstream Yangtze sub-basins, as well as in the Hai, Huai, and Songhua river basins (Figure S8). Maintaining food production at the level of today while avoiding water pollution in all Chinese rivers and streams seems infeasible. A certain minimum amount of N is needed to maintain the current crop yield, which contributes to water pollution in 16–18% of the streams under all scenarios. These results are in agreement with the finding of Schulte-Uebbing et al.^3^ and Yu et al.^2^ They show that under current production conditions, nutrient use efficiencies need to be higher than 80%, to ensure surface water quality without compromising the food production requirements, which is beyond what is considered as feasible.^33^ To achieve both clean water and food security, current dietary patterns of the population need to be reconsidered, and system-changed strategies need to be implemented.^34−36^ For example, effective spatial planning of livestock production is proposed to dramatically reduce N losses to the environment from the livestock sector.^30,37^ A new system for recycling human waste to replace traditional wastewater treatment plants could also be considered.^2^ For example, it has been demonstrated that decentralized wastewater treatment technologies have the potential in increasing the recycling rate of wastewater.^38,39^

### 3.3 Uncertainties and Sensitivity Analyses

The main uncertainties identified in this modeling study are associated with (1) the uncertainties in the chosen threshold of N concentration for setting the regional N boundaries and (2) the uncertainties in the modeling approach, parameters, and modeling inputs.

We used the modeled DIN concentrations to set the regional N boundaries for China. DIN is the most bioavailable form that causes negative environmental impacts on surface waters.^28,40^ We quantified that DIN loads dominate (i.e., exceed DON loads) in around 84% of the streams.^8^ Nevertheless, our approach of using DIN introduces uncertainties in our derived regional N boundaries. This is because particulate N and organic N could also contribute to the associated negative impacts on water systems.^41,42^ The chosen threshold of 1 mg/L for DIN could potentially underestimate the required N reductions needed to keep N use within the regional N boundaries. This is because 1 mg/L is also commonly used as a threshold for TN concentration, which implies that keeping the modeled DIN concentration below 1 mg/L does not mean that the TN concentration will also be below 1 mg/L. This suggests that the N boundary for fulfilling the actual TN concentration below 1 mg/L may have to be stricter than our estimates.

**Figure 6:**
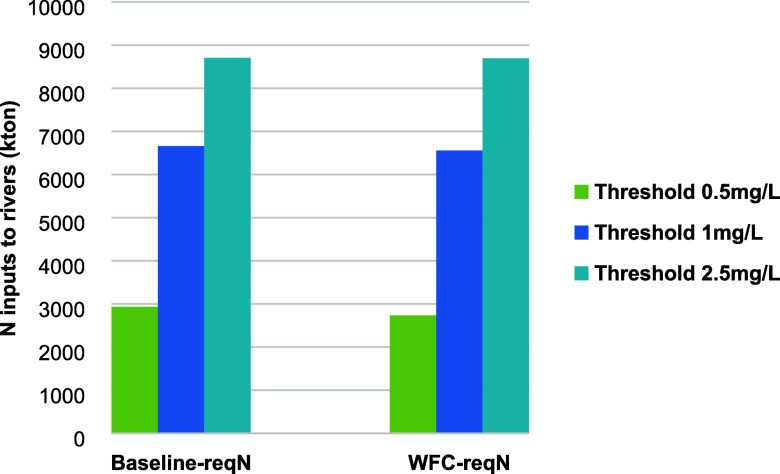
Calculated maximum levels of N inputs to rivers (kton/year) as China’s share in a planetary boundary (PB) aimed at water quality levels not exceeding thresholds in the Baseline-reqN and WFC-reqN scenarios. The chosen thresholds are 0.5, 1, and 2.5 mg/L for DIN concentrations. Baseline refers to the situation in 2012; WFC refers to Whole Food Chain nutrient management and recycling. The “reqN” scenarios refer to scenarios that quantify the regional N boundaries by applying our bottom-up approach (Section 2).

To address the associated uncertainties, we analyze the sensitivity of our modeled regional N boundaries within the range of the thresholds of N concentration (0.5 mg/L to 2.5 mg/L) based on literature reviews,^2,4,27−29^ i.e., 0.5 mg/L, 1 mg/L, and 2.5 mg/L (Figure 6). The range of the national N boundary for China based on the surface water quality threshold of 0.5–2.5 mg/L is around 2.9–8.7 Tg (6.6 Tg for 1 mg/L) for the Baseline and 2.7–8.7 Tg (6.5 Tg for 1 mg/L) for WFC. De Vries et al.^4^ applied the 1 mg/L and 2.5 mg/L values as critical thresholds for TN concentrations to quantify the planetary boundary for N. The associated PB for N is around 33% higher for the 2.5 mg/L threshold than for 1 mg/L. Our study also indicates a 30% increase in the associated N boundary for China when comparing the 1 mg/L threshold to the 2.5 mg/L threshold. This implies that the derived regional boundaries for N are rather sensitive to the critical threshold of N concentration. We also notice that the sensitivity in the estimated N inputs to rivers is in particular high in the range of 0.5–1 mg/L compared to 1.0–2.5 mg/L (Figure 6). This phenomenon is in line with the concept of “reversibility and hysteresis” in the theory of “ecological threshold,”^43^ which in this case suggests that restoring the water quality to comply with a stringent threshold requires more effort than reducing the original exceedance of the pollutants. As other large-scale studies for regional N boundaries,^2−4,7^ we used the uniformed threshold for all river sections. For future research, the spatially explicit N threshold in surface water reflecting the spatial variability in environmental tolerance of local water systems should be incorporated, which could further improve our spatially explicit estimates of the regional boundary for N. Research progresses have been made by emerging studies on deriving regional-specific thresholds for nutrient pollution in Chinese rivers.^44−47^ However, these studies are scattered in time and space with small-scale field studies for small rivers or certain river sections of large basins. A complete and comparable set of region-specific N thresholds for rivers in China is still lacking.

Uncertainties also exist in the modeling structure, inputs, and parameters. Chen et al.^20^ and Chen et al.^8^ discussed these aspects in detail. Here, we summarize the main uncertainties that could affect the derived regional N boundaries. First, the particulate nitrogen is not included in our model structure, which hampers the estimation of the regional N boundary based on TN concentration. Several studies indicate that TN criteria may be more appropriate than DIN criteria for toxic ecological impacts induced by DIN on water systems.^28,48,49^ We addressed the associated uncertainties via the different thresholds chosen thresholds above. Second, we used the modeled 30-years average gridded (0.5° x 0.5°) discharge over the 1970–2000 period from the VIC hydrological model which was run at the global scale at 0.5°.^22,23,50^ VIC has been validated using daily observed records of the streamflow for 1,557 river monitoring stations showing a realistic representation of the observed conditions for stations globally. Although the average discharge is not changing dramatically around the current year 2012, this difference in the reference year may influence the estimation of nutrient concentrations, thus influencing the derived regional N boundaries. Third, the global calibrated constant value that presents the biological process of in-stream retentions of N for Chinese rivers may lead to a conservative estimate of in-stream retentions of DIN in our model.^8,51,52^ This suggests that our modeled DIN concentrations may be overestimated, which results in potential underestimation of the derived regional N boundaries. Moreover, the modeled inputs are mainly based on year 2012 rather than year 2020. By comparing the main drivers (e.g., fertilizer N use and manure N applied to land) between 2012 and 2020, we observed a generally smooth changes in these drivers, which implies that the analysis of year 2012 still has both societal and scientific significance for current N management (see details in S2.2).

### 3.4 Model Strengths and Policy Implications

We realize that important steps had been made toward improved understanding the environmental impacts of N pollution on water systems and associated pollution management by abundant local scale studies. These studies have generally provided more detailed spatial and temporal level of pollution status and associated source attribution by two types of approaches: site-level measurements, stable isotopes combined with statistical analysis^53−59^ (e.g., Bayesian analysis and 1-D mathematical equation for environmental capacity calculation) or small-scale process-based models^60−65^ such as SWAT^62^ and GBNP.^61^ However, providing such detailed-level assessments for the whole of China is theoretically possible but practically not feasible yet, as it requires substantial input data and measurements for calibration and validation. A comprehensive assessment of regional N boundary for the whole of China is needed for a consistent comparison of N boundary across multiple regions and to support both holistic and region-specific policy-making for China. Below, we elaborate the advantages of our modeling approach and usefulness in supporting associated policy-making.

First, our study is strong in providing comprehensive modeling assessments of the regional N boundary based on surface water quality for whole China incorporating the main processes within N cycles and supporting associated nutrient management by providing sufficient spatial level of details for administrative policy-making. This is due to (1) the unique spatial levels of our modeling units (e.g., polygons as intermediate units to bridge biophysical and administrative scales), (2) the state-of-art input data sets (e.g., county statistics of 2238 counties and WWTP database consisting of 4204 WWTPs), (3) largely process-based modeling approach with validation (e.g., main processes of nutrient cycling), (4) newly developed bottom-up approach (e.g., accounting for complex response of N inputs to N concentrations), and (5) a novel integration of nutrient modeling systems (e.g., NUFER county for food production with MARINA 3.0 for water quality). The extended discussions of above five aspects are presented in S2.2.

Second, our multiscale spatially explicit modeling results could provide more policy-relevant insights compared to traditional biophysical scale modeling. Our study contributes to various policies including the “River Chief System,”^66^ “Agriculture Green Development (ADG),”^67,68^ and “Spatial planning of industrialized livestock production.”^71,72^ In the year 2016, the “River Chief System” was issued,^66^ aiming to establish the river chiefs nationwide to support the implementation of the “10-Point Water Plan.”^69^ A river chief, as the name suggests, will be responsible for the management and protection of watercourses as well as the coordination of related administrators for pollution prevention. We believe that our presented results can support the decision-making for various stakeholders in this regulation. For instance, river chiefs and associated county mayors could develop policy plans using county-specific N boundaries and reduction targets (as quantified in our study), in line with the regional N boundaries of larger basins.

In 2021, China launched a new national policy focusing on promoting Agriculture Green Development (AGD).^67,68^ The AGD aims to transform the current intensive resource use and high environmental cost of agriculture to a new agriculture system with high productivity, high resource use efficiency, and low environmental impact. Our study contributes to this policy in the following aspects. First, one of the main themes of AGD is to reduce agricultural fertilizer use and thus reduce the nutrient pollution of water systems. Our study provides scientific insights into how to balance nutrient use in agriculture and nutrient pollution in water systems. Second, one of the five main actions proposed by ADG is integrating animal–crop production and increasing the recycling rate of manure, which is in line with the measures of the WFC scenario included in our study. The findings contribute to an improved understanding of the effectiveness of the associated proposed actions on surface water quality. Moreover, researchers^30,37,70^ and Chinese government^71,72^ have proposed spatial planning of industrialized livestock production to reduce negative impacts of nutrient surpluses to environments. It shows that the N concentration threshold in surface water quality could be the most stringent criterion for N pollution management.^3^ In this aspect, our derived spatially explicit N boundaries could contribute to the national livestock reallocation plan, for instance by identifying the areas and associated environmental capacities to receive livestock and areas where livestock production has to be reduced due to water pollution.

### 3.5 Main Findings

We have developed a new spatially explicit approach to quantify the maximum allowable inputs of N to Chinese rivers and streams as China’s share in planetary boundaries for N. Despite the uncertainties, we consider our study as an important step toward a better understanding of how to keep N use within boundaries to restore China’s surface water quality. Our results lead to the following main findings:

- The maximum N inputs to Chinese rivers are around 6.5 Tg N based on the situation in the year 2012.
- There are large regional differences in N boundaries. Maximum N inputs to rivers are relatively high for regions such as the North China Plain where food production is intensive and relatively low for regions such as the Downstream Zhujiang where urbanization rates are high.
- Assuming the overapplication of synthetic fertilizers and animal manure is reduced, 45% and 76% of the rivers could meet the water quality thresholds in the Baseline and WFC for the year 2012, respectively.
- Comparing the Baseline and WFC scenarios indicates that the current levels of nutrient use efficiencies in food production need to be largely improved for reducing water pollution.
- A comparison of “water quality first” and “food production first” scenarios indicates that trade-offs between water quality and food production exist in 2–8% of the streams, which may put 7–28% of crop production at stake.
- Maintaining food production at the level of today while avoiding water pollution in China does not seem possible. This could be a reason to reflect and reconsider current dietary patterns of the population.

## Supporting Information Available

Detailed description for MARINA 3.0 model, quantifications of the developed bottom-up approach, study area, scenario descriptions, and extended descriptions of the results and associated discussions; the overview of the nutrient modeling framework of MARINA 3.0 (Model to Assess River Inputs of Nutrients to seAs) (Figure S1); example of the routing procedure to calculate required reductions for the in-stream load (kton) per grid cell (Figure S2); overview of (sub-)basins included in this study (Figure S3); calculated required reduction of N inputs to rivers (kton/year) to ensure that N concentrations do not exceed the PB threshold (1 mg/L) for the Baseline-reqN and WFC-reqN scenarios (Figure S4); dissolved inorganic nitrogen (DIN) inputs to rivers (kton) in the Baseline (A) and Whole Food Chain (WFC) management scenario (B) for the year 2012 on multiple scales (sub-basin, 0.5#x30a;grid, county, and polygon) (Figure S5); the proportion of surface water course’ DIN > 1 mg L-1 (a,c,e,g) to the total streamlines (”polluted surface water(%)”) and proportion of population living in areas (Figure S6); the normalized current N inputs to rivers against the derived boundary on 414 provincial scale (Figure S7); locations of streams with trade-offs for Baseline and WFC scenarios (Figure S8); overview of the sources of the model inputs for nutrient inputs to rivers that are changed in the WFC (Whole Food Chain management) scenario and their associated differences between Baseline and WFC (Table S1); crop uptake factors of crop specifies for different scenarios (Table S2); overview of eight scenarios (Table S3) (PDF)
