# Supplementary material for: Keeping Nitrogen Use in China within the Planetary Boundary Using a Spatially Explicit Approach
Source: Environ Sci Technol. 2024 May 23;58(22):9689–700. doi: 10.1021/acs.est.4c00908 (PMC11155250; doi:10.1021/acs.est.4c00908)
Supplement: Supplementary file 1 — es4c00908_si_001.pdf [file es4c00908_si_001.pdf]

Supporting information

Keeping nitrogen use in China within the planetary boundary using a spatially-explicit approach

Xi Chen[1,2,3*], Maryna Strokal[2], Michelle T.H. van Vliet[4],Ling Liu[1], Zhaohai Bai[1], Lin Ma[1,5*], Carolien Kroeze[6]

[1] Key Laboratory of Agricultural Water Resources, , Hebei Key Laboratory of Soil Ecology, Center for Agricultural Resources Research, Institute of Genetics and Developmental Biology, Chinese Academy of Sciences, 286 Huaizhong Road, Shijiazhuang 050021, China

[2] Water Systems and Global Change Group, Wageningen University & Research, Droevendaalsesteeg 4, Wageningen, 6708 PB, The Netherlands

[3] Institue of Urban Environment, Chinese Academy of Sciences, 1799 Jimei Road, Xiamen 361021, China

[4] Department of Physical Geography, Utrecht University, P.O. Box 80.115, 3508 TC Utrecht, The Netherlands

[5] State Key Laboratory of Pollution Control and Resource Reuse, School of the Environment, Nanjing University, Nanjing 210023, Jiangsu, China.

[6] Environmental Systems Analysis Group, Wageningen University & Research, Droevendaalsesteeg 4, Wageningen, 6708 PB, The Netherlands

Correspondence to: Xi Chen (xchen@iue.ac.cn); Lin Ma (malin1979@sjziam.ac.cn)

Supporting information consists of the following sections:

S1 Model description.. 2

S1.1 Nutrient inputs to rivers 5

S1.2 In-stream nutrient retention and routing 8

S1.3 The rationale of the bottom-up approach.. 10

S1.4 The quantifications of the bottom-up approach 10

S1.5 Management options to keep N use in agriculture within the regional N boundary.... 14

S2 Supplementary results 18

Reference.. 29

The supplementary information (31 pages) contains the detailed description of MARINA 3.0 model, quantifications of the developed bottom-up approach, the supplementary results and discussions, three supplementary tables, and eight supplementary figures.

S1 Model description

We developed a new bottom-up approach to quantify and meet the spatially-explicit nitrogen (N) boundary in China for complying with the surface water quality threshold. MARINA 3.0 (Model to Assess River Inputs of Nutrients to seAs) model framework has been modified and applied for this end. The model framework is based on three existing models: MARINA 1.0 (*M*odel to *A*ssess *R*iver *I*nputs of Nutrients to se*A*s) [4], NUFER (*NU*trient flows in *F*ood chains, *E*nvironment and *R*esources use) [5], the VIC hydrological [7,8], and VIC-RBM water temperature model [7-9,11] The MARINA 1.0 (we also refer to this model as MARINA-Nutrients, China-1.0) model provides the basis for modeling nutrient inputs to rivers and their exports to seas. Outputs of NUFER and VIC are used as inputs to our MARINA model. NUFER provides model inputs for 2338 counties in China in 2012 [6,12]. Examples of such inputs are synthetic fertilizers and animal manure applications on land. VIC provides the hydrological data such as total runoff on a 0.5° × 0.5° grid for quantifying the soil retention processes [7,8].

The previous versions of the MARINA models (1.0 and 2.0) for nutrient flows in China run on the sub-basin scale and quantify the river export of nutrients at the outlets of these sub-basins [4,10,13]. In these earlier versions, the in-stream surface water pollution was not modelled due to their relatively coarse representations. There are three main improvements in MARINA 3.0 compared to previous versions of the model. The first improvement is in the quantification of nutrient inputs to rivers. We downscale the sub-basin approach by developing a multi-scale framework (polygon approach) to bridge the administrative scales (e.g. county) with biophysical scales (e.g. grid and sub-basin). Moreover, we distinctly improve the modelling for point sources (see Chen et al. [2]). The second improvement is in the quantification of in-stream nutrient pollution[3]. Based on nutrient inputs to rivers, we account for retentions of nutrients (in-stream retentions, retentions by dams and reservoirs, and consumptive water use), and transports of nutrients by the river network (upstream to downstream influences). The retention processes have been downscaled from sub-basin to grid scale, and the in-stream retention now takes a process-based modelling approach as opposed to a calibrated approach in earlier version of MARINA. Finally, we validated the model by both river sections and river mouths (while previous versions focussed validation only on river mouths). Following the improvements of MARINA 3.0, we modified the model to incorporate the developed bottom-up approach (back-routing module) to quantify the regional N boundaries.

Figure S1 presents an overview of the modeling framework of our MARINA 3.0 (we also refer to this model as MARINA-Nutrients, China-3.0). It consists of two main parts to quantify in-stream surface water quality of nutrients and the associated sources for China. The first part is a multi-scale nutrient model to quantify annual nutrient inputs to rivers for the year 2012 [2]. The second part takes the model outputs of the nutrient inputs to rivers from the first part and accounts for in-stream nutrient retentions and transport of nutrients by the river network (i.e. routing procedure to account upstream to downstream nutrient accumulation) [3]. The model in the end can produce in-stream nutrient concentration, river export of nutrients in the river mouth, and the associated source attributions by nutrient forms. The nutrient forms include dissolved inorganic (DIN, DIP) and dissolved organic (DON, DOP) nitrogen and phosphorous. In this study, we only focus on the nutrient form of DIN and its in-stream nutrient concentrations.

MARINA 3.0 is described by the following three sections: nutrient inputs to rivers (section S1.1), in-stream nutrient retention (section S1.2), and in-stream nutrient concentrations (section S1.3). Below, we describe the original MARINA 3.0 model by each model part, with an emphasis on how the model/ model inputs are modified for this study. For the further details of the MARINA 3.0 model, we refer to Chen et al [2] and Chen et al [3].

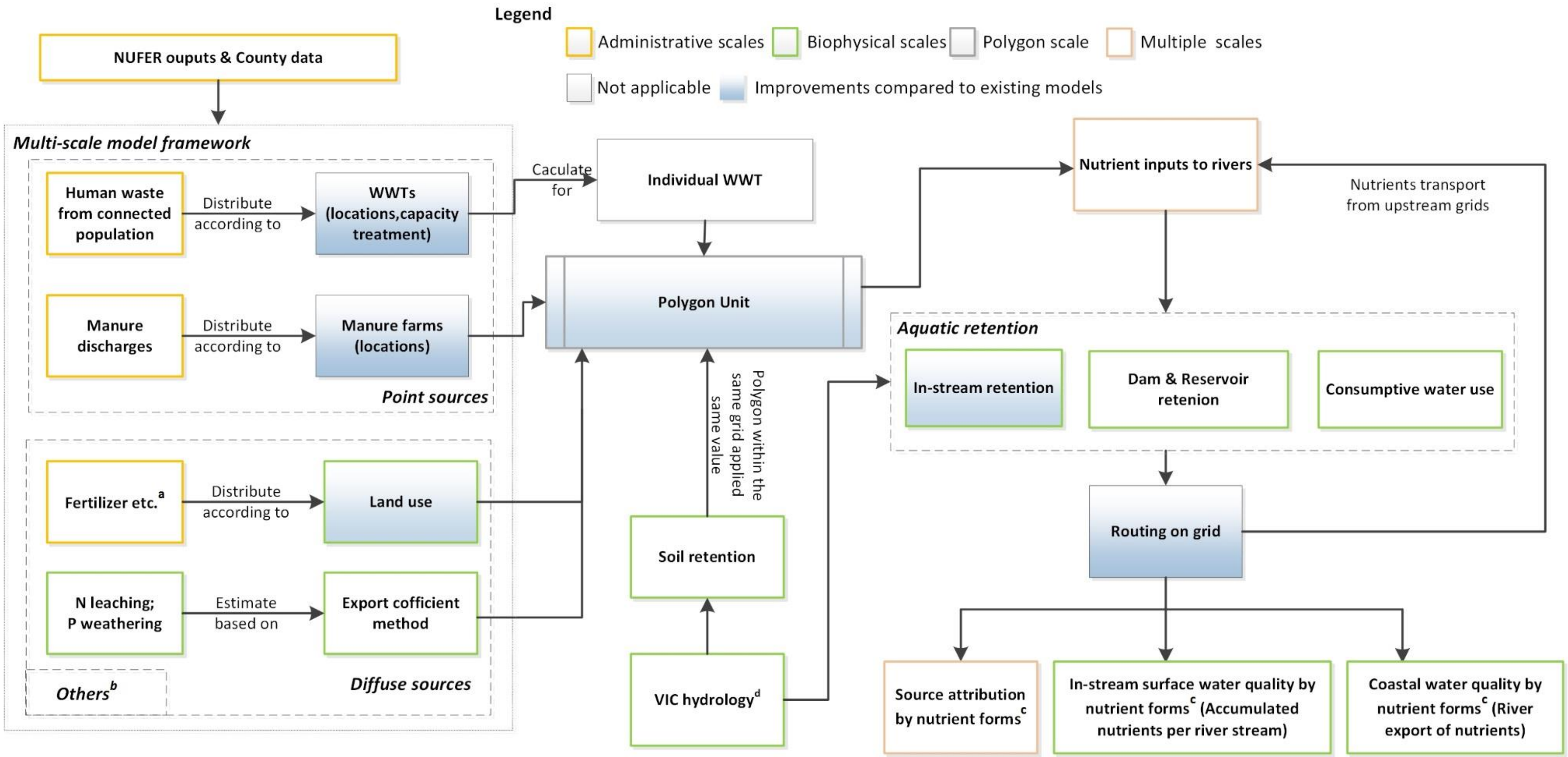

Figure S1. *The overview of the nutrient modelling framework of MARINA 3.0 (Model to Assess River Inputs of Nutrients to seAs). a: This box includes manure application on land, biological N_2 fixation, atmospheric N deposition, crop export via harvesting and animal grazing, and human waste applied on land from human waste resulted from popiulation that is not connected to sewage systems. b: Others include human waste from unconnected population that is directly discharged to water bodies (without treatment). c: Nutrient forms include dissolved inorganic (DIN, DIP) and dissolved organic (DON, DOP) nitrogen and phosphorous. d: The data includes for instance runoff,discharge and water temperature. This paper focuses on the DIN model outputs. Administrative scales refer to county or provincial scale; Biophysical scales refer to a grid of* 0.5 ° x 0.5 ° *, sub-basin and basin scales; A polygon unit is the intermediate scale of the biophysical and administrative scales, resulting from the intersection of a county and a 0.5 °grid. The figure is adapted from Chen et al* [2] *and Chen et al* [3]*. NUFER is short for NUtrient flows in Food chains, Environment and Resources use model* [5, 6]*. VIC is short for* Variable Infiltration Capacity *model* [7-9]*. WWT is short for waste water treatment plant. The blue color in boxes indicates the improvements compared to previous MARINA 1.0 and 2.0 models* [4, 6, 10]*, which are summarized in Chen et al* [2] *and Chen et al* [3]*. 'Multiple scales' in the legend include polygon,* 0.5 ° *grid, (sub-)basin, and county*

S1.1 Nutrient inputs to rivers

The nutrient inputs to rivers are quantified based on the multi-scale nutrient model published by Chen et al [2]. The model quantifies inputs of N and phosphorus (P) to rivers by the source on both biophysical (e.g. sub-basin, grid) and administrative scales (e.g. county). The advantages and improvements of the multi-scale modeling compared to the previous versions of MARINA 1.0 and 2.0 [4, 10] are summarized in Chen et al [2] and Chen et al [3]. In this study, we use the model outputs on a 0.5° x 0.5° grid of nutrient inputs to rivers in the following equation [2]:

$$\boldsymbol{RStotal_F} = \boldsymbol{RS_{dif_F}} + \boldsymbol{RS_{pnt_F}} + \boldsymbol{RS}_{other\,s_F} = \left(\boldsymbol{FE_{ws,F}} \cdot \boldsymbol{WS_{dif_F}} + \boldsymbol{RSdif_{EC_F}}\right) + \boldsymbol{RS_{pnt\_ma,F}} + \boldsymbol{RS_{pnt\_con,F}} + \boldsymbol{RS}_{others_F} \quad \text{(Eq.S1)}$$

Where, $RStotal_F$ is the total inputs of the nutrient form (F) from land to streams by 0.5° grid (kg year^{-1}). The nutrient form in this study refers to DIN. These DIN inputs in rivers and streams resulting from point (RS_{pnt_F}), diffuse (RS_{dif_F}) and other sources (RS_{others_F}) (kg year^{-1}). Diffuse sources (RS_{dif_F}, kg year^{-1}) include explicit land sources (WS_{dif_F}), which are corrected via the export fraction ($FE_{ws,F}$, 0-1) that includes the retention of nutrients in soil and parameterized export processes ($RSdif_{EC_F}$, kg year^{-1}). Explicit land sources are synthetic fertilizer use, and animal manure that are applied on land, human waste that is applied on land from rural and urban populations that are not connected to sewage systems, biological N_2 fixation, and atmospheric N deposition. Parameterized export processes reflect inputs to streams such as the leaching of organic matter that is not applicable for DIN. Point sources ($RS_{\boldsymbol{pnt_F}}$) include direct discharges of animal manure to streams ($RS_{pnt\_ma,F}$, kg year^{-1}) and human waste emitted from wastewater treatment plants (WWTPs) ($RS_{pnt\_con,F}$, kg year^{-1}). RS_{others_F} is the direct discharge of the nutrient form (F) to streams from human waste that is not connected to WWTPs in a polygon (kg year^{-1}).

For each source (e.g. RS_{dif_F}), there are associated equations and model parameters for the quantifications. For the full overview of sources of the input data and model parameters, equations for each source category, and approach to convert from different scales (e.g. polygon to the grid) we refer to the SI in Chen et al (2019). Table S1 presents the overview of the sources and the associated changes of the model inputs for nutrient inputs to rivers that are changed in the WFC (Whole Food Chain management) scenario compared to the baseline of the year 2012 [2].

Table S1. *Overview of the sources of the model inputs for nutrient inputs to rivers that are changed in the WFC (Whole Food Chain management) scenario and their associated differences between Baseline and WFC. For the original data sources of the WFC scenario, we refer to Jin et al* [1]*. S1 refers to management strategy 1, i.e. increasing soil fertility; S2 refers to management strategy 2, i.e. abandoning discharge of manure and increasing recycling of manure; S3 refers to improved livestock manure management with low ammonia emission; S4 includes new systems to recycle human excretion and food waste.*

*: The associated changes differ among counties and here only presents the national changes.

Abbreviation of model inputs	Description	Spatial scale	Reference of the input sources for the year 2012	Changes between the WFC scenario and the baseline scenario for the year 2012 on a national scale (% change)*	Associated management strategies in WFC that leads to the changes in model inputs
**Diffuse sources**					
$WSdif_{fe,ant,N}$	Synthetic fertilizers applied on land (kg year^{-1})	County	[6, 12]	Decreased by 71%	S1
$WSdif_{ma,ant,N}$	Animal manure applied on land (kg year^{-1})	County	[6, 12]	Increased by 180%	S2
$WSdif_{dep,ant,N}$	Atmospheric N deposition on agricultural areas (kg year^{-1})	County	[6, 12, 14, 15]	Decreased by 40%	S3
$WSdif_{fix,ant,N}$	Biological N_2 fixation by crops (kg year^{-1})	County	[6, 12]	Increased by 12%	S1
$WSdif_{ex,N}$	Nutrient export via crop harvesting and animal grazing (kg year^{-1})	County	[6, 12]	No change	NA
$WSdif_{hum,ant,N}$	Human excreta applied from unconnected population to sewage (kg year^{-1})	County	NA**	Increased by 230%	S4
$WSdif_{dep,nat,N}$	Atmospheric N deposition on natural areas (kg year^{-1})	County	[6, 12, 14, 15]	Decreased by 30%	S3

$\boldsymbol{WSdif_{fix,nat,N}}$	Biological N_2 fixation from the natural area (kg $year^{-1}$)	Grid	[16, 17]	No change	NA
**Point sources**					
$RS_{pnt\_ma,DIN}$	Manure discharges of DIN (kg $year^{-1}$)	County	[6, 12]	Decreased by 100%	S3
$\boldsymbol{RS_{pnt\_con,DIN}}$	DIN from human waste emitted from centralized WWTPs	Individual WWTP	[2]	Decreased by 100%	S4
**Others**					
$\boldsymbol{RS_{others_{DIN}}}$	human waste that is directly discharged to rivers (untreated) from the population without sewage connections (kg $year^{-1}$).	County	[2, 4]	Decreased by 100%	S4

S1.2 In-stream nutrient retention and routing

In this section, we explain the retentions and routing of nutrients along the stream network that is included in the model framework. The retentions of nutrients in the stream network are quantified by two parts: the 'sub-grid' retention and the 'grid-stream' retention. The followings describe the quantifications of these two parts and the routing (i.e. transport process) of nutrients. For the extended descriptions and sources of model inputs, we refer to the SI of Chen et al [3].

Nutrient inputs to rivers are first transported by lower-order streams to higher-order streams (Strahler order) [18]. The large river presented explicitly by the streamlines (as streamlines of Figure 6 in the main text) in our model is derived based on the DDM30 flow direction map [19] on a 0.5° × 0.5° grid, which is defined as 'grid-stream' in our study. In addition to the explicit streamlines by the DDM30 (Strahler order of six or higher), there are lower-order streams that are not represented by the derived streamlines. We define these lower-order rivers (Strahler order from 1 to 5) which are not reflected by streamlines as a 'sub-grid' network followed by Beusen et al [20]. As such, nutrient inputs to rivers are first entering lower-order streams (from 1 to 5) and are retained by 'sub-grid' before entering 'grid-stream' (Strahler order 6 or higher) represented by the grid streamline network.

After being retained and transported by the 'sub-grid' network, nutrients entering the 'grid-stream' will be transported (routing) further based on the flow direction network of DDM30 on a 0.5° × 0.5° grid scale [19] to next 'grid-stream'. Each grid cell receives nutrient inputs from the local cell ($RStotal_{F_{local},i}$ in Eq.S2) and from upstream located grid cells ($IS_{F_{upstream}}$ in Eq.S2). Nutrients in a grid cell are then retained ($FE_{riv.F,i}$, Eq.S2) before entering the next grid cell. The same procedure holds for the next downstream located grid cell. After the retentions, the in-stream nutrients are transported to the next grid cell and the same procedures apply. The calculation continues until nutrients reach the river mouth or until the flow in a certain grid cell does not drain to the next grid cell (i.e. inland rivers):

$$\boldsymbol{IS_{F,i} = (RStotal_{F_{local},i} + IS_{F_{upstream}}) \cdot FE_{riv_{F,i}}} \quad \text{(Eq.S2)}$$

$$\boldsymbol{RStotal_{F_{local},i,} = \left(RS_{dif_F} + RS_{pnt_{ma},F} + RS_{others_F}\right) * L_{F_{subgrid},i} + RS_{pnt\_con,F}} \quad \text{(Eq.S3)}$$

$$\boldsymbol{FE_{riv_{F,i}} = (1 - L_F) \cdot (1 - D_F) \cdot (1 - \mathrm{FQrem})} \quad \text{(Eq.S4)}$$

Where, $IS_{F,i}$ refers to the in-stream nutrient accumulation load by nutrient form (F) in a grid of 0.5° (kg year^{-1}). $RStotal_{F_{local},i}$ refers to the total input of nutrient form from a grid of 0.5 °(the local grid cell i, i.e. $RStotal_F$ in Eq.S1 after correcting for sub-grid retentions that entering the higher order streamlines of the grid (kg year^{-1}). These inputs originate from activities (point and diffuse sources) in their own grid cells. $IS_{F_{upstream}}$ refers to the input of nutrient form F from upstream grid cells of 0.5° (kg year^{-1}). $FE_{riv_{F,i}}$ refers to the export fraction of nutrient form (F) in high-order streams that are represented by streamline i of 0.5°grid cell (0-1). The export fraction includes three retention processes. L_F is in-stream retention within the river network, channels, river bed sediments, and floodplains (0-1). D_F is the nutrient retention within constructed reservoirs and behind dams (0-1). *FQrem* is the removal from streams through water removal for irrigation and other human needs (0-1). All these retentions are calculated on a 0.5° grid cell and we refer to the details of the quantifications in Chen et al [3] (see SI section S1.2 in that paper). $L_{F_{subgrid},i}$ refers to nutrient retentions in a 'sub-grid' network (i.e. lower-order streams) of grid i (0-1). The parameterization of these sub-grid networks is followed by accepted geomorphic principles [21, 22]. The parameterization of low-order streams ('sub-grid) and quantifications of in-stream retentions by the sub-grid network are described in Chen et al [3] SI section S1.2.

The in-stream nutrient concentration is then calculated as follows:

$$\boldsymbol{N_{a,i}} = \frac{\boldsymbol{IS_{F,i}}}{\boldsymbol{Q_i}} \quad \text{(Eq.S5)}$$

Where, $\boldsymbol{N_{a,i}}$ is the in-stream concentration of dissolved inorganic or organic N in grid cell i (mg/L), depending on the nutrient form (F) of $\boldsymbol{IS_{F,i}}$ (the in-stream nutrient accumulated load in grid i of 0.5°, kton/year) in Eq.1. $\boldsymbol{Q_i}$ is the modeled annual river discharge in grid cell i of 0.5° from the VIC hydrological model (L/year) [7, 8, 23].

S1.3 The rationale of the bottom-up approach

The original MARINA 3.0 model calculates the in-stream nutrient concentration following the sequence of (a) calculations of nutrient inputs to rivers, (b) retentions of nutrients along the river network (by rivers, dams and consumptive water use), (c) river transport (routing/accumulation according to flow direction) and (d) in-stream concentration. We can understand the bottom-up approach as the back calculations as opposed to the original model. It starts from the gap of the in-stream nutrient concentration Q_i and 1 mg/L threshold and then converts the gap of the concentration to accumulated N load ($IS_{N,gap,i}\ in\ Eq.S6$) (associated with steps d and c in the original model). Afterward, the up-stream to downstream impact (routing) is corrected to get the required reduction of in-stream accumulated N load ($IS_{N,rdc,i}\ in\ Eq.S7$) (associated with steps c and b in the original model). Finally, the conversion from accumulated N load to the required reduction of nutrient inputs to rivers ($RStotal_{DIN,rdc,i}\ in\ Eq.S17$) (associated with step a in the original model).

S1.4 The quantifications of the bottom-up approach

Below we demonstrate the associated quantifications of Step 1 to Step 4 in section 2.1.2 of the main text.

Step 1. Quantifying the gap of accumulated in-stream load per grid cell

The reduction gap of accumulated N load per grid cell is calculated as:

$$\boldsymbol{IS_{N,gap,i} = (N_{a,i} - N_{t,i}) \cdot Q_i} \quad \text{(Eq.S6)}$$

Where, $\boldsymbol{IS_{N,gap,i}}$ refers to the gap of the in-stream (IS) accumulated load of dissolved inorganic nitrogen (N) in grid cell i (kton/year) between the current in-stream load (modeled, kton/year) and the targeted in-stream load (kton/year, based on the surface water quality threshold of 1m/L multiplying with discharge). $\boldsymbol{N_{a,i}}$ is the in-stream concentration of dissolved inorganic N in grid cell i (mg/L). This concentration is calculated from the in-stream accumulated load of dissolved inorganic N in grid cell i (kton/year) divided by the river discharge of that grid cell (L/year). $\boldsymbol{N_{t,i}}$ is the critical threshold of N in streams (i.e. 1 mg/L) and is assumed to be uniform for all

grid cells. $\boldsymbol{Q_i}$ is the modeled annual river discharge (L/year) in grid cell i from VIC hydrological model [7,8,23]. Grid cell resolution is 0.5°x0.5°.

Step 2. Quantifying the required reduction of accumulated in-stream load per grid

The complex relationships between N inputs to rivers and N in-stream concentrations differ in space. The reduction of N in-stream load on upstream grid cells influence the reduction of downstream located grid cells depending on the river network, flow direction, and associated retentions. In this step, the calculated gap of accumulated N load is routed along the river network to correct for the influence of the upstream grid cell reduction on downstream grid cell. After the routing, the actual required reduction of in-stream N load per grid cell is quantified.

The routing starts from the upstream grid cells to the downstream grid cells according to the flow direction [19]. The reduction of accumulated load (in-stream load) in upstream cells lower the required reduction load in downstream-located grid cells. We illustrate this process with an example of 3 grid cells (*Figure S2*). Grid cell 1 is the headwater grid. Water from this grid flows into grid cell 2, which subsequently flows into grid cell 3. The routing starts from the headwater grid (i.e. grid 1 in *Figure S2*). First, the gap of the accumulated N load ($\boldsymbol{IS_{N,gap,i}}$) in surface water for each grid cell is calculated from step 1. The gap of accumulated N load is needed to be corrected by upstream impacts to get the actual required reduction of the in-stream load of local grids. The procedure is as follows: the gap of accumulated N load from the headwater grid cell (e.g. grid cell 1 in *Figure S2*) is reduced to meet the surface water quality threshold (i.e. equal to the required reduction of the in-stream load of the headwater grid cell). Next, the required reduction of in-stream load from the headwater grid cell is applied to retentions of the next grid cell and then routed to the next grid cell. We use the reduction of N in-stream load transported by upstream grid cells ($\boldsymbol{IS_{N,upstream,i}}$ in Eq.S8) to calculate the required reduction of in-stream load of local-specific grid cells. The required reduction of the in-stream load of a certain grid cell is equal to the gap of accumulated N load of that grid cell minus the reduction of N in-stream load transported by upstream grid cells or is equal to 0, depending on the reduction of N transport by upstream grid cells (accounted for retentions) smaller or greater than the current gap of N accumulated load of that grid cell. In this way, we correct the compensation effects (i.e. the reduction load of upstream grid cells decrease the reduction needed from downstream cells) of reductions from upstream-located grid cells. The associated steps are as follows (Eq S7 to Eq S16):

For grid 1,

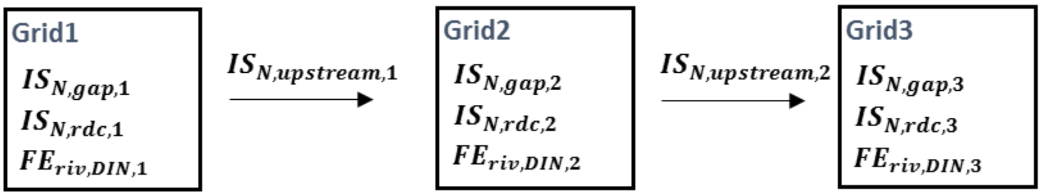

Figure S2. An example of the routing procedure to calculate required reductions for the in-stream load (kton) per grid cell of 0.5°.

$$\boldsymbol{IS_{N,rdc,1} = IS_{N,gap,1}} \tag{Eq.S7}$$

$$\boldsymbol{IS_{N,upstream,1} = IS_{N,gap,1} * FE_{riv,DIN,2}} \tag{Eq.S8}$$

Where, $\boldsymbol{IS_{N,rdc,1}}$ refers to the required reduction of the in-stream accumulated N load (kton) of grid cell 1 to meet the threshold of surface water quality. Grid 1 is the headwater grid cell. $\boldsymbol{IS_{N,upstream,1}}$ refers to N transport by grid cell 1 (kton) to the next grid cell 2. $\boldsymbol{FE_{riv,DIN,2}}$ refers to the river export fraction of dissolved inorganic N (DIN) for grid 2 (0-1), including in-stream retentions, retentions by dams and reservoirs, and removal by consumptive water use in this grid cell 2 (see S1.2). Below the parameters in the equations (Eq.S9 to Eq.S16) are the same as Eq.S7 and Eq.S8, but with differences in the grid cell number.

Equations for the headwater grid cell 1 that drains towards the downstream grid cell 2 are:

If $\boldsymbol{IS_{N,upstream,1} > IS_{N,gap,2}}$,

$$\boldsymbol{IS_{N,rdc,2} = 0} \tag{Eq.S9}$$

$$\boldsymbol{IS_{N,upstream,2} = IS_{N,upstream,1} \cdot FE_{riv,DIN,3}} \tag{Eq.S10}$$

If $\boldsymbol{IS_{N,upstream,1} < IS_{N,gap,2}}$,

$$\boldsymbol{IS_{N,rdc,2} = IS_{N,gap,2} - IS_{N,upstream,1}} \tag{Eq.S11}$$

$$\boldsymbol{IS_{N,upstream,2} = (IS_{N,upstream,1} + IS_{N,rdc,2}) \cdot FE_{riv,DIN,3}} \tag{Eq.S12}$$

Equations for grid 2 flowing to further downstream located grid 3 are:

If $IS_{N,upstream,2} > IS_{N,gap,3}$,

$$IS_{N,rdc,3} = 0 \quad \text{(Eq.S13)}$$

$$IS_{N,upstream,3} = IS_{N,upstream,2} \cdot FE_{riv,DIN,4} \quad \text{(Eq.S14)}$$

If $IS_{N,upstream,2} < IS_{N,gap,3}$,

$$IS_{N,rdc,3} = IS_{N,gap,3} - IS_{N,upstream,2} \quad \text{(Eq.S15)}$$

$$IS_{N,upstream,3} = (IS_{N,upstream,2} + IS_{N,rdc,3}) \cdot FE_{riv,DIN,4} \quad \text{(Eq.S16)}$$

The “if” equations (Eq. S9 to Eq. S16) include two different situations. The first situation (Eq.S9 and Eq.S13) is that the required reduction of the accumulated N load to meet the water quality threshold is zero for a grid cell. For that grid cell, we do not need to reduce N to meet the water quality threshold. This can happen when the required reduction of the accumulated N load from the upstream grid cells ($IS_{N,upstream,i}$) fulfills (larger than) the gap of the accumulated load of the downstream located grid cell ($IS_{N,gap,i}$), the required reduction of in-stream load from this local-specific grid cell is not needed (i.e. 0). Otherwise (Eq.S11 and Eq.S15), the required reduction of the in-stream load of this local grid cell ($IS_{N,rdc,i}$) is equal to the gap of the accumulated load of this grid cell minus the reduction of N in-stream load transported by upstream grid cells.

The procedure is continued along the routing process until the required reduction of the in-stream load over all grid cells along the river network has been calculated.

Step 3 Quantifying the required reduction of N inputs to rivers

We calculate the required reduction for N inputs to streams using the required reduction for accumulated in-stream loads (see Step 2). This is done as follows::

$$RStotal_{DIN,rdc,i} = \frac{IS_{N,rdc,i}}{FE_{riv,DIN,i}} \quad \text{(Eq.S17)}$$

Where, $RStotal_{DIN,rdc}$ refers to the required reduction of nutrient inputs to rivers for grid cell i (kton/year). $FE_{riv,DIN,i}$ refers to the river export fraction of nutrient form DIN for grid cell i

(0-1), including in-stream retentions, retentions by dams and reservoirs and removal by consumptive water use (see section S1.2).

Step 4 Quantifying the boundary of N inputs to rivers

The regional boundary for N inputs to rivers is equal to the current N inputs to rivers per grid cell minus the required reduction of N inputs to river per grid cell:

$$\boldsymbol{N_{boundary,i} = RStotal_{DIN,local,i} - RStotal_{DIN,rdc}} \quad \text{(Eq.S18)}$$

Where, $\boldsymbol{N_{boundary\_n}}$ refers to the regional N boundary of N inputs to rivers (kton/year) of grid cell i, based on the surface water quality (N concentration) threshold. $\boldsymbol{RStotal_{DIN,local,i}}$ refers to the total inputs of DIN to rivers of a certain grid cell i (kton/year). $\boldsymbol{RStotal_{DIN,rdc}}$ is explained in Eq.S17.

The regional boundaries for N inputs to rivers on 0.5˚ grid scale are further converted to county, sub-basin and national scale by the multi-scale modelling approach developed by MARINA 3.0. The multi-scale approach takes around 13000 polygons for China as the intermediate modelling units, which are resulted from the intersection between Chinese counties and 0.5˚ grids. Using the polygon as the intermediate scale, the gridded regional boundaries for N could first allocate to the polygons located within the same grid based on the ratio of the total N inputs to rivers of the polygon to total N inputs to rivers of the grid (for calculation of N inputs to rivers on polygon scale see details in Chen et al.[2]). The regional N boundaries on polygon scale can upscaling to county, to sub-basin and to nation based on the associated univocal spatial relations that links them to the corresponding grid, county, and sub-basin (details in Chen et al.[2]).

S1.5 Keeping nitrogen use in agriculture within the regional boundary

We consider management options for agriculture to keep N use within the derived regional boundary, i.e. fulfilling the required reduction for each region (e.g. grids, counties). We identify the targeted reduction sources from agriculture, which include synthetic fertilizer applied on agricultural land and manure applied on agricultural land. Two rules are considered when reduce the agricultural N use:

1. the use of synthetic fertilizers and animal manure should first ensure crop production and then be reduced to meet the water quality threshold.

2. first ensure water quality meets the threshold via reducing agricultural N inputs to land (synthetic fertilizers and animal manure); crop requirements could be compromised.

Below we describe how we quantify the required reduction of N use from land based on above two rules.

We introduce the uptake factor on the grid scale (converted from county to grid-scale, explained below) to ensure crop production requirements when N inputs are reduced. Therefore, the maximum reduction allowed in N inputs in agriculture is calculated as follows:

$$\boldsymbol{N}_{\boldsymbol{rdc,ag_{potential},i}} = \boldsymbol{WSdif}_{\boldsymbol{fe,ant,N}} + \boldsymbol{WSdif}_{\boldsymbol{ma,ant,N}} + \boldsymbol{WSdif}_{\boldsymbol{dep,ant,N}} + \boldsymbol{WSdif}_{\boldsymbol{fix,ant,N}} + \boldsymbol{WSdif}_{\boldsymbol{hum,ant,N}} - \boldsymbol{Uptake}_{\boldsymbol{county,i}} \cdot \boldsymbol{WSdif}_{\boldsymbol{ex,N}} \qquad \text{(Eq.S19)}$$

Where, $\boldsymbol{N}_{\boldsymbol{rdc,ag_{potential},i}}$ refers to the maximum allowed reduction of N inputs to agricultural land (kton/year) while ensuring food production in grid cell i. $\boldsymbol{WSdif}_{source,ant,N}$ is the N inputs to land from different sources (kton/year); subscript 'ant' refers to the inputs originating from the agricultural area. $\boldsymbol{WSdif}_{source,ant,N}$ includes synthetic fertilizer use ($\boldsymbol{WSdif}_{\boldsymbol{fe,ant,N}}$, kton/year), animal manure that is applied on land ($\boldsymbol{WSdif}_{\boldsymbol{ma,ant,N}}$, kton/year) and human waste that is applied on land from rural and urban populations disconnected from sewage systems ($\boldsymbol{WSdif}_{\boldsymbol{hum,ant,N}}$, kton/year), biological N_2 fixation ($\boldsymbol{WSdif}_{\boldsymbol{fix,ant,N}}$, kton/year), atmospheric N deposition ($\boldsymbol{WSdif}_{\boldsymbol{dep,ant,N}}$, kg kton/year), $\boldsymbol{WSdif}_{\boldsymbol{ex,N}}$ is the nutrient export via crop harvesting and animal grazing (kton/year), $\boldsymbol{Uptake}_{\boldsymbol{county,i}}$ is the uptake factor of crop production originally on the county scale, which represents the N inputs required (kton) to grow 1 kton of crop (based on the crop species, see Table S2). We applied the area-weighted approach to downscale the uptake factor of counties to grid cell level. The area-weight approach is based on the proportion of the arable area per polygon to the area of arable land of the county that polygon is located in (see details in Chen et al.[2]).

Table S2. *Crop uptake factors of crop specifies for different scenarios.*

	BAU[1]	WFC
Cereals	1.4	1.11[24, 25]
Beans	0.015	0.015[1]

Potatoes	1.8	1.8[1]
Oil crops	1.4	0.96[26]
Sugar crops	1.4	1.4[1]
Other crops	1.7	1.11[26]
Pasture	1.4	1.40[1]
Vegetable	2.0	1.57[27]
Fruit	2.0	1.57[27]

The result of Eq.S19 provides insights on the maximum allowed reduction of N inputs to agricultural land (kton/year) while ensuring food production over all grid cells. In steps 1-3 above, we calculate the required reduction of N inputs to rivers. Eq.S17 quantifies the required reduction of N inputs to rivers. We use this information to back-calculate how much reduction of N inputs to agricultural land is required for all grid cells (Eq.S20 below). As a result, we have two values of the required reduction of N inputs to agricultural land:

- Value 1: the maximum allowed reduction of N inputs to agricultural land while ensuring food production (Eq. S19 above)
- Value 2: the required reduction of N input to agricultural land calculated from the required reduction of N in rivers (Eq.S20 below), based on the water quality threshold (Steps 1-4 above)

Next, we compare these two values. If value 2 is larger than value 1 ($\boldsymbol{N}_{\boldsymbol{rdc,ag_{potential},i}}$ in Eq.S19), then we correct the actual reduction of N inputs from land to the maximum reduction of N inputs allowed (Eq.S21 below). This correction is only applied to the 'food security first' scenarios (i.e. first ensure crop production then reduce water pollution, see details in section 2.2 of main text) and implies that not all required reduction of N inputs to rivers calculated from the above steps could be fulfilled due to the crop production requirement. For the 'water quality first' scenario (i.e. first ensure water quality regardless of crop production, see details in section 2.2 of main text), Eq.S21 below is not applicable. The following equations describe these processes:

$$\boldsymbol{WSdif}_{\boldsymbol{DIN,rdc,i}} = \frac{\boldsymbol{RStotal}_{\boldsymbol{DIN,rdc,i}}}{\boldsymbol{FE}_{\boldsymbol{ws,DIN,i}} \cdot \boldsymbol{L}_{\boldsymbol{DIN\_subgrid,i}}} \quad \text{(Eq.S20)}$$

Where, $\boldsymbol{WSdif}_{\boldsymbol{DIN,rdc,i}}$ refers to the required reduction of N inputs from land (kton/year) of grid cell i. $\boldsymbol{FE}_{\boldsymbol{ws,DIN,i}}$ is the export fraction of nutrient form (DIN) entering rivers of grid cell i, correcting for the nutrient retentions by soil (0-1). $\boldsymbol{L}_{\boldsymbol{DIN\_subgrid,i}}$ refers to the fraction of DIN entering to rivers, correcting for the retentions in lower-order streams (0-1).

If $\boldsymbol{WSdif}_{\boldsymbol{DIN,rdc,i}} > \boldsymbol{N}_{\boldsymbol{rdc,ag_{potential},i}}$,

$$\boldsymbol{WSdif}_{\boldsymbol{DIN,rdc,i}} = \boldsymbol{N}_{\boldsymbol{rdc,ag_{potential},i}} \quad \text{(Eq.S21)}$$

This step is to ensure the required reduction of N inputs from land (kton/year) do not compromise food production. In this way, we corrected the excess required reduction of N inputs from land to ensure crop production for 'food security first' scenarios (section 2.2 below). This means that for these grid cells the surface water quality threshold could not be met due to the food production requirement.

S2 Supplementary results

S2.1 Figures and tables

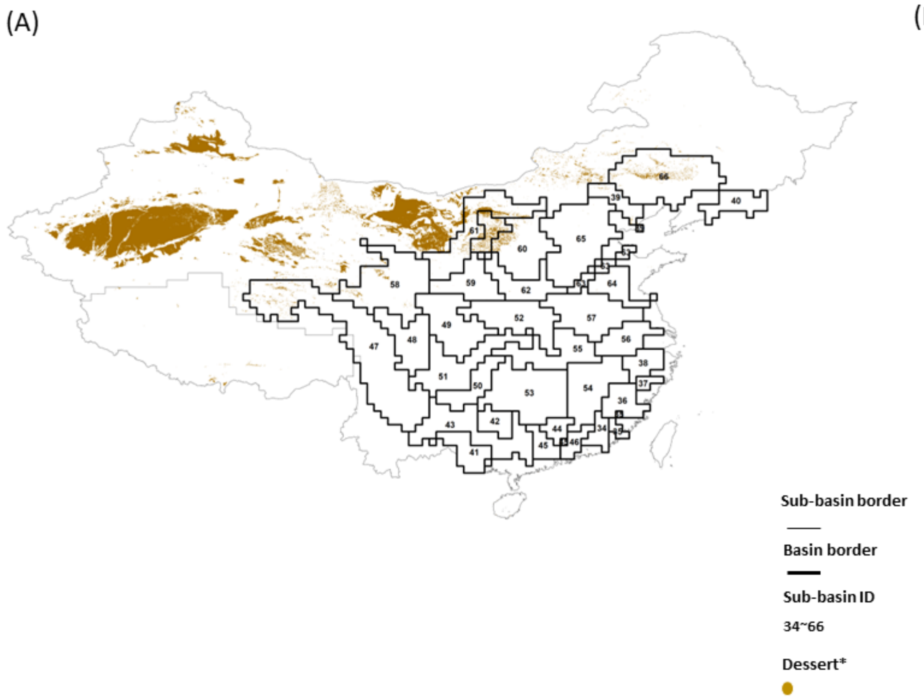

(B)

Sub-basin ID	Name	Basin
	Other Inland River	
34	Hanjiang	
35	Jiulong	
36	Menjiang	
37	Qujiang	
38	Fuchun	
39	Luan	
40	Yalu	
41	Yujiang	Pearl River
42	Liujiang	Pearl River
43	Xijiang	Pearl River
44	Beijiang	Pearl River
45	Delta zhujiang	Pearl River
46	Dongjiang	Pearl River
47	Jinsha	Yangtze
48	Min	Yangtze
49	Jialing	Yangtze
50	Wu	Yangtze
51	Upstream Yangtze	Yangtze
52	Han	Yangtze
53	Dongting	Yangtze
54	Poyang	Yangtze
55	Middlestream Yangtze	Yangtze
56	Delta Yangtze	Yangtze
57	Downstream Yangtze	Yangtze
58	Lanzhou	Huang He
59	Wehe	Huang He
60	Longmen	Huang He
61	Toudaogual	Huang He
62	Huayuankou	Huang He
63	Delta Yellow	Huang He
64	Huai	
65	Hai	
66	Liao	

Figure S3 *Overview of (sub-)basins included in this study. (A) Maps of (sub-)basins. (B) The names of sub-basins and basins in this study. *Dessert is identified according to the land-use data (1×1 km grid) [28] of year 2010.*

Table S3. *Overview of eight scenarios. Two of them are the reference scenarios (the Baseline year 2012 and Whole Food Chain (WFC)1). Each reference scenario has three alternatives (in total six). Alternative scenario 1 focuses on the Regional N boundary (regN). Alternative scenario 2 focuses on Measure – 'water quality first' (wq1st). Alternative scenario 3 focuses on Measure 'food security first' (food1st).*

Scenarios	***Baseline***	***Whole Food Chain (WFC) management***
Reference scenarios for the year 2012	• *N use as in the year 2012*	• *Improve nutrient use efficiencies through whole food chains via various management strategies for N use in the year 2012 (see SI-S4 strategies shown in Figure 2)*<br>• *The WFC management implies that synthetic fertilizer use is decreased, animal manure use is increased, no direct discharge of manure, atmospheric N deposition is decreased, recycled human waste is increased and human waste from WWTPs is decreased compared to the Baseline scenario*
Regional N boundary (regN)	***Baseline-regN:***<br>• *Based on N concentration in the year 2012 and 1mg/L threshold*<br>• *Apply the bottom-up approach (Section 2) to derive N inputs to rivers as a regional N boundary*	***WFC-regN:***<br>• *Based on N concentration in WFC and 1mg/L threshold*<br>• *Apply the bottom-up approach (Section 2) to derive N inputs to rivers as a regional N boundary*
Measure – 'water quality first' (wq1st)	***Baseline-wq1st:***<br>• *Quantify the required reduction from fertilizer and manure applied for meeting the derived regional N boundary of the baseline*<br>• *Crop production could be at stake*	***WFC-wq1st:***<br>• *Quantify the required reduction from fertilizer and manure applied for meeting the derived regional N boundary of WFC*<br>• *Crop production could be at stake*

(Table to be continued in next page)

Table S3. *Continued.*

Measure – 'food security first' **(food1st)**	***Baseline-food1st:***<br>• *Quantify the required reduction from fertilizer and manure applied for meeting the derived regional N boundary of the baseline*<br>• *If the crop production is at stake, correct the needed reduction of fertilizer and manure to secure crop production*	***WFC-food1st:***<br>• *Quantify the required reduction from fertilizer and manure applied for meeting the derived regional N boundary of WFC*<br>• *If the crop production is at stake, correct the needed reduction of fertilizer and manure to secure crop production*

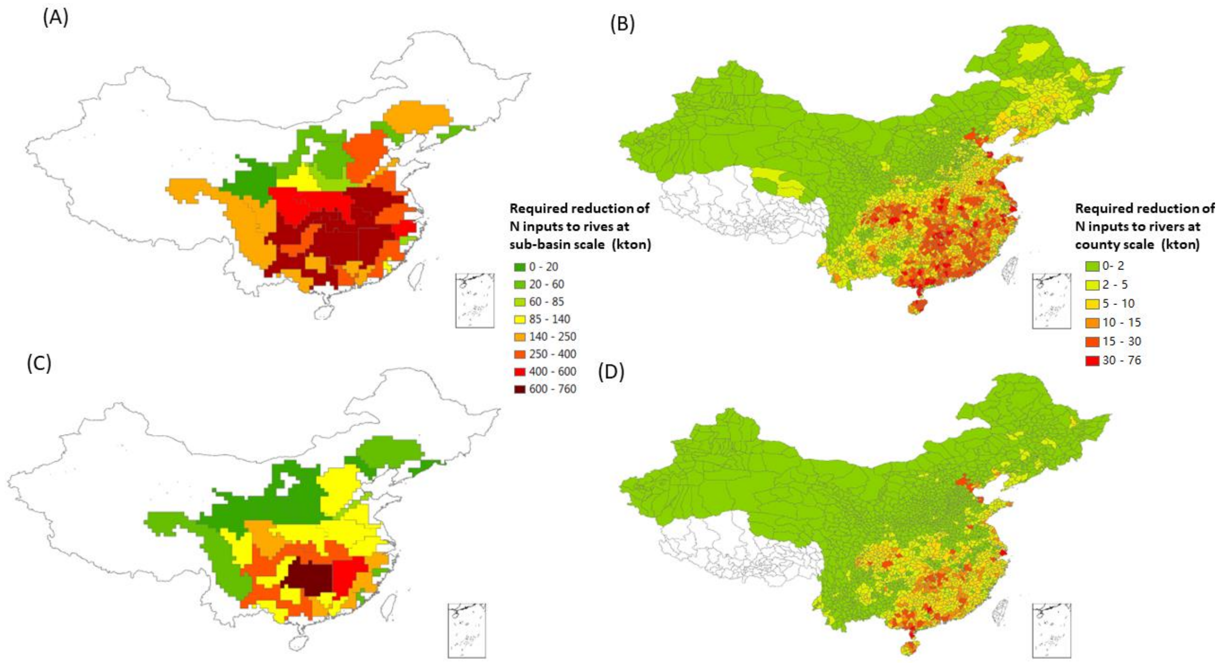

Figure S4. *Calculated required reduction of N inputs to rivers (kton/year) to to ensure that N-concentrations do not exceed the PB threshold (1 mg/L) for the Baseline-reqN and WFC-reqN scenarios: (A). Required reduction of N inputs to rivers for sub-basins for the 2012 Baseline; (B). Required reduction of N inputs to rivers for counties for the 2012 Baseline; (C). Required reduction of N inputs to rivers for sub-basins for the Whole Food Chain (WFC) management scenario; (D). Required reduction of N inputs to rivers for counties for the WFC scenario. 'reqN' refers to scenarios that quantify the regional N boundary by applying bottom-up approach (section 2). The study area of sub-basins are presented in Figure S3 of SI.*

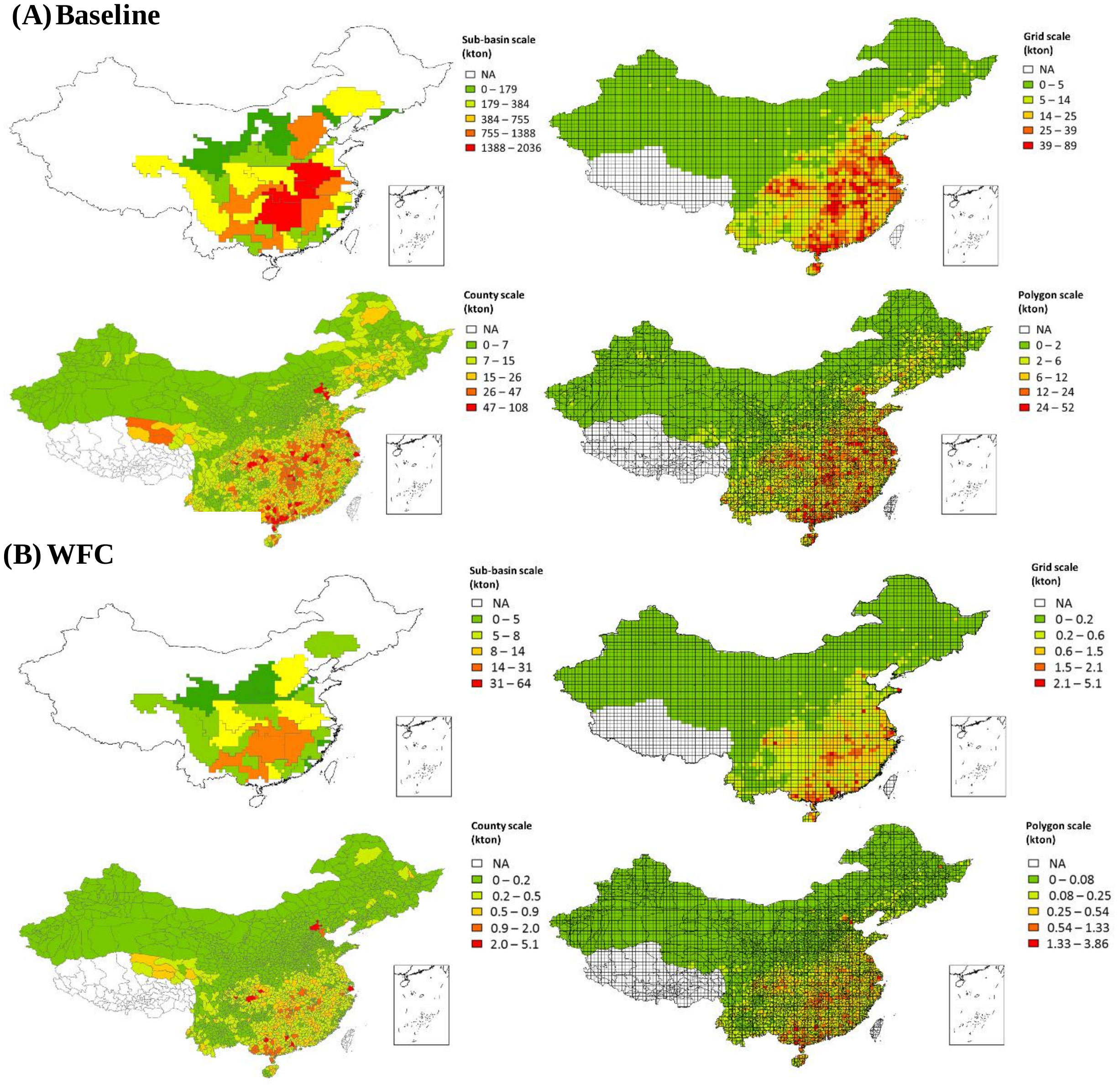

Figure S5. *Dissolved inorganic nitrogen (DIN) inputs to rivers (kton) in the Baseline (A) and Whole Food Chain (WFC) management scenario (B) for the year 2012 on multiple scales (sub-basin, 0.5° grid, county and polygon). NA indicates regions for which detailed input data are Not Available.*

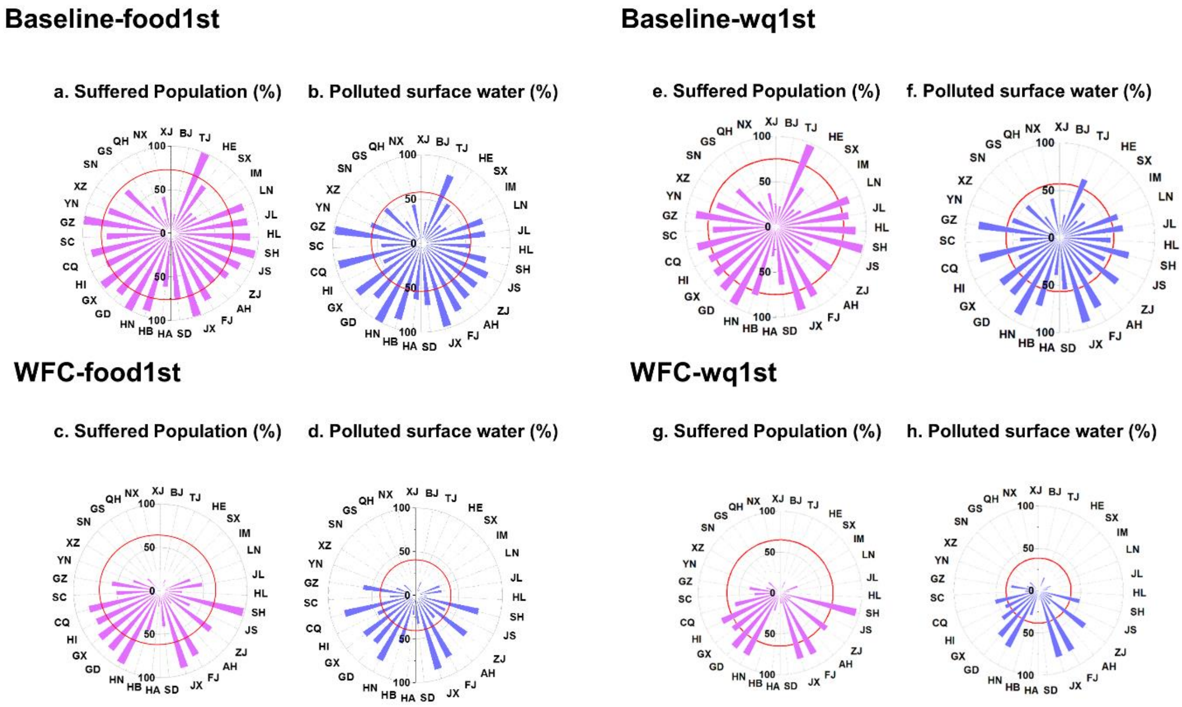

Figure S6. *The proportion of surface water course' DIN > 1 mg L_{-1} (a,c,e,g) to the total streamlines ('Polluted surface water(%)') and proportion of population living in areas where water quality does not meet the requirements (bd,f,h) to total population at provincial level ('Suffered Population(%)')) under different bottom-up scenarios. Baseline refers to 2012 baseline. WFC refers to whole-food-chain management based on 2012 scenario. The red cycles in the graphs refer to the national average value of the associated parameters under baseline or WFC. Baseline refers to baseline of year 2012. WFC refers to whole-food-chain management based on 2012 scenario [1]. Baseline-food1st and WFC-food1st refers to the scenarios that first ensure crop needs and then reduce the extra fertilizer and manure for meeting water quality threshold. Baseline-wq1st and WFC-wq1st refer to the scenarios that first ensure water quality without considering crop needs (details in section 2). The abbreviations of the outlier of the cycle (e.g. 'NX') represents the official abbreviations of Chinese provinces.*

S2.2 Extended discussion

Comparisons between Baseline and WFC of derived boundaries and required reduction of N inputs to rivers

The differences of the derived boundaries between the Baseline and WFC scenarios are due to the combined effects of the different spatial distribution of current N inputs to rivers, the associated retention and transport by the river network. This results in different gaps between the current N in-stream concentration and its threshold, the associated different gaps of in-stream accumulated load and thus the associated N boundaries. However, they are not distinctly different, particularly when aggregated to larger scale (sub-basin, county) as indicated by Figure 3 (main text). This is due to the larger ranges of the boundaries for these scales. The analysis on 0.5° grid scale shows that around 30% and 12% of grids the changes in the boundaries between these two scenarios are more than 20% and 50%, respectively.

Although the spatial distribution of N inputs to rivers could influence on the calculated boundaries, the differences of N inputs to rivers between the Baseline and WFC scenarios could lead to larger differences in the required reduction of N inputs to rivers (Figure S4) than the calculated boundaries. This is because within a (sub-)basin when the concentrations of most of streams exceed or are close to the threshold, the calculated boundaries could be very closed to the theoretical capacity of pollution load for each stream (assuming all streams reaching 1mg/L and back-calculate the N inputs to rivers as the theoretical capacity of pollution load for each stream). The required reduction of nutrient inputs to rivers (the gap between current nutrient inputs to rivers and the boundary) are routing along the river network to correct the impact of upstream grid cell reduction on downstream grid cells (step 2 in section 2 and section S1.4). Therefore, the required reduction of nutrient inputs to rivers are largely influenced by the spatial distribution of original nutrient inputs to rivers of two scenarios.”

Comparisons with study of Yu et al.[29]

Yu et al. [29] presented the provincial scale ratios which are based on the provincial N inputs to rivers normalized against the provincial critical pollution threshold (ranging from 0 – 10, Figure 4a in Yu et al[29]) instead the actual value of the provincial N boundary. It made the direct comparison of the derived boundaries difficult. We add the Figure S7 for the comparison by deriving above provincial scale ratios based on our results. The ratios indicate the extent of the exceedance of current N inputs to rivers by critical boundaries and the associated required reduction of N inputs to meet the boundaries. On national scale, a 64% reduction of N inputs to rivers is needed based on Yu et al.[29], while our study presented a 65% required reduction of N inputs to rivers. The spatial distribution of the ratios is generally in line with their estimates. Some of provinces of which the ratios are relatively higher or lower. This may due to different modelling approach and data sources in quantifying the associated boundaries and N inputs to rivers.

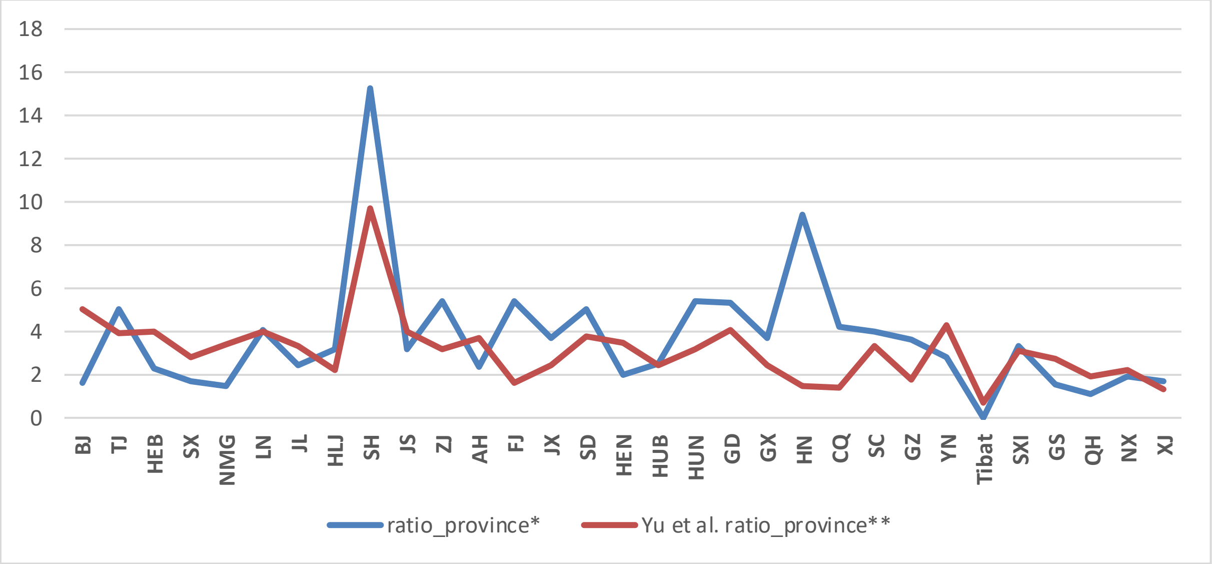

Figure S7. *The normalized current N inputs to rivers against the derived boundary on provincial scale; the results are based on this study and Yu et al.[29]. 'ratio_province* refers to the results of this study. 'Yu et al. ratio_povince**' refers to the results of Yu et al.[29] and the value is visual determined from Figure4a of Yu et al.[29].*

Comparisons between 'water quality first' and 'food security first' scenarios

By comparing the 'water quality first' and 'food security first' scenarios, we identify the trade-offs between water quality and food production. In the manuscript, we stated that 'The comparisons indicate that the trade-offs between water quality and food production exist in 2-8% of the streams, which put 7-28% of the crop production at stake, depending on the Baseline or WFC scenario'. Here, the streams with tradeoffs are identified as the streams where 'food production first' scenario remained polluted (i.e. concentrations exceed 1mg/L) while 'water quality first' scenarios become unpolluted (i.e. concentraions below 1mg/L). And we further quantify the N requirements of crop production (see details in Section S1.5) for these streams divided by the national total N requirements of crop production to get the percentage of crop production influenced. Figure S8 shows the locations of the streams with trade-offs for Baseline and WFC scenarios. Noting that the Baseline has fewer streams with trade-offs than WFC, this is due to the reduction in Baseline lead to limited streams become unpolluted (i.e. concentrations still exceed 1mg/L after reductions). If we compare the required reduction of N inputs to agricultural land (Eq.S20) minus the maximum allowed reduction of N inputs to agricultural land (Eq.S22), we get that 35% and 18% of streams are with tradeoffs (i.e. the required reduction of agricultural N use for surface water quality larger than the maximum allowed reduction of N inputs to agricultural land based on food security) for Baseline and WFC, respectively.

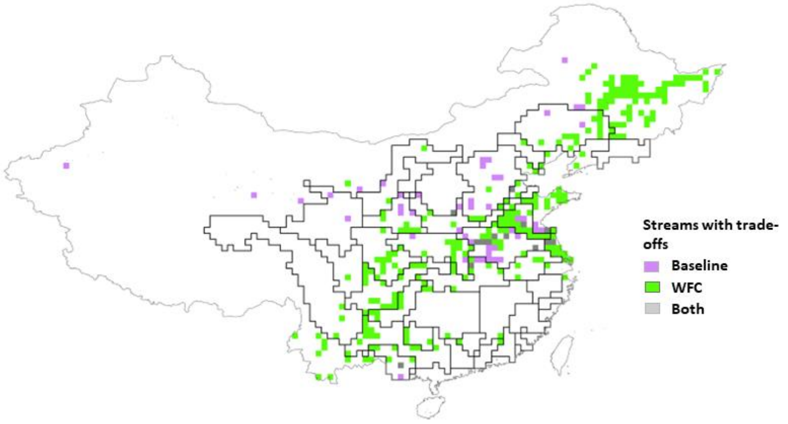

Figure S8*. Locations of streams with trade-offs for Baseline and WFC scenarios. The streams with tradeoffs are identified as the streams where 'food production first' scenario remained polluted (i.e. concentrations exceed 1mg/L) while 'water quality first' scenarios become unpolluted (i.e. concentraions below 1mg/L). WFC refers to whole-food-chain management scenario based on year 2012. Baseline refers to baseline of year 2012. Both refers to streams with trade-offs in both Baseline and WFC.*

Comparisons between year 2012 and year 2020s

We used the data of year 2012 because these datasets are so far most complete, consistent and well-evaluated model inputs for our modelling systems, consisting of county statistics of 2238 counties for whole China[9], NUFER-county results[11, 30], a unique wastewater treatment plant (WWTP) database consisting of 4204 WWTPs[20, 63, 64] and measurements of 155 monitoring stations across China[8, 65, 66]. As other large-scale studies (e.g. modelling for whole China) using the datasets for year 2010s[29-31], the data availability of most current years (e.g. year 2020s) are constrained by many reasons (e.g. consistency, availability and data for validation). Our aim is to further improve and update the input datasets to implement a consistent and well-evaluated modelling application in the near future. Despite the limitations of not using the most current data inputs, we still consider our study is useful in terms of (1) improvements of **modelling approach** to quantify regional N boundaries that directly link the N inputs to N concentrations; (2) **scientific insights** gained of how to balance N use in agriculture and N pollution in surface water and (3) the potential for **future analysis** provided by the developed model approach.

Additionally, we also analysed to what extent the presented results of year 2012 are meaningful to represent current situation (year 2020s). By comparing the trend of the main drivers (including fertilizer N use, livestock production (in stock) and manure N excretions and manure N applied to land) between 2012 and 2022, we observed a generally smooth changes in these drivers. The comparisons between year 2012 and 2022 indicate that a 30% of reduction in fertilizer N use, a 4% increase of livestock production (in stock, animal number), a 2% increase of manure N excretions and a 0.5% increase of manure N applied to land on national scale for China[32]. This is because the period of 1980 to 2010s is the most dramatically changing period for China's agriculture transition [33]. From 2010s to 2020s, China has implemented a series of policies to regulate the impact of agriculture development on water quality, including 'Zero growth in fertilizer'[34], ''10-Point Water Plan' [35] and 'Agriculture Green Development'[36]. Therefore, year 2010s possibly, represent the worst situation of water pollution by agricultural production. It is meaningful to understand the mechanisms behind, the pollution caused at multiple spatial levels, as well as the synergies and trade-offs between water quality and food production, and the solutions in future. Additionally, our WFC management scenario that incorporated state-of-art N management measures could somehow represent a better N management version of current years. Based on above, the analysis of year 2012 still has both societal and scientific significance for current N management.

Model strength

Our model is strong in providing comprehensive modelling assessments of the regional N boundary based on surface water quality for whole China incorporating the main processes within N cycles and support associated nutrient management by providing sufficient spatial level of details for administrative policy-making. This is due to (1) the unique spatial levels of our modelling units, (2) the state-of-art input datasets, (3) largely process-based modelling approach with validation, (4) newly developed bottom-up approach and (5) a novel integration of nutrient modelling systems.

We used a novel formalized framework for multi-scale modelling of nutrient pollution in water systems[2,3]. This modelling approach takes polygons as intermediate modelling units to bridge the traditional modelling scale of water quality on the biophysical scales (e.g. grid, sub-basin) and human drivers or policy-making on the administrative scales (e.g. city, county). This is particular helpful to support policy-relevant questions (commonly on administrative scales) to regulate nutrient pollution in Chinese rivers (biophysical scale). Additionally, we used state-of-art modelling datasets, including county statistics of 2238 counties for whole China[12], NUFER-county results[1,6], a unique wastewater treatment plant (WWTP) database consisting of 4204 WWTPs[2,37,38] and measurements of 155 monitoring stations across China[3,39,40]. Our model is largely process-based, taking into account the main processes of nutrient cycling including nutrient inputs by different sources from land, nutrient inputs to rivers, the biochemical processes within the river network and the transport of nutrients by river network. The model is evaluated and our modelling estimates correspond well with measurements of pollution levels and water quality classes. Moreover, compared to current large-scale modelling studies for deriving regional N boundaries, our approach is improved by explicitly including the complex relationships between N inputs and concentrations throughout the river network, which we argue is essential for defining a meaningful spatially-explicit N boundary. Finally, with a novel integration of our water quality model (MARINA 3.0)[2,3] with NUFER-county model[1,6] allows us to explore and assess the impacts of agricultural management not only on water quality but also on food security.

Reference

(1) Jin, X.; Bai, Z.; Oenema, O.; Winiwarter, W.; Velthof, G.; Chen, X.; Ma, L. Spatial planning needed to drastically reduce nitrogen and phosphorus surpluses in China’s agriculture. *Environ. Sci. Technol.* **2020**, *54* (19), 11894-11904.

(2) Chen, X.; Strokal, M.; Van Vliet, M. T.; Stuiver, J.; Wang, M.; Bai, Z.; Ma, L.; Kroeze, C. Multi-scale modeling of nutrient pollution in the rivers of China. *Environ. Sci. Technol* **2019**, *53* (16), 9614-9625.

(3) Chen, X.; Strokal, M.; van Vliet, M. T.; Fu, X.; Wang, M.; Ma, L.; Kroeze, C. In-stream surface water quality in China: A spatially-explicit modelling approach for nutrients. *Journal of Cleaner Production* **2022**, *334*, 130208.

(4) Strokal, M.; Kroeze, C.; Wang, M.; Bai, Z.; Ma, L. The MARINA model (Model to Assess River Inputs of Nutrients to seAs): model description and results for China. *Sci. Total. Environ.* **2016**, *562*, 869-888.

(5) Ma, L.; Ma, W.; Velthof, G.; Wang, F.; Qin, W.; Zhang, F.; Oenema, O. Modeling nutrient flows in the food chain of China. *J. Environ. Qual.* **2010**, *39* (4), 1279-1289.

(6) Wang, M.; Ma, L.; Strokal, M.; Ma, W.; Liu, X.; Kroeze, C. Hotspots for Nitrogen and Phosphorus Losses from Food Production in China: A County-Scale Analysis. *Environ. Sci. Technol.* **2018**, *52* (10), 5782-5791.

(7) Van Vliet, M. T.; Sheffield, J.; Wiberg, D.; Wood, E. F. Impacts of recent drought and warm years on water resources and electricity supply worldwide. *Environ. Res. Lett* **2016**, *11* (12), 124021.

(8) Van Vliet, M. T.; Wiberg, D.; Leduc, S.; Riahi, K. Power-generation system vulnerability and adaptation to changes in climate and water resources. *Nat. Clim. Change.* **2016**, *6* (4), 375.

(9) Yearsley, J. R. A semi‐Lagrangian water temperature model for advection‐dominated river systems. *Water Resources Research* **2009**, *45* (12).

(10) Wang, M.; Kroeze, C.; Strokal, M.; van Vliet, M. T.; Ma, L. Global change can make coastal eutrophication control in China more difficult. *Earth's Future* **2020**, *8* (4), e2019EF001280.

(11) Van Vliet, M.; Yearsley, J.; Franssen, W.; Ludwig, F.; Haddeland, I.; Lettenmaier, D.; Kabat, P. Coupled daily streamflow and water temperature modelling in large river basins. *Hydrology and Earth System Sciences* **2012**, *16* (11), 4303-4321.

(12) Data Center for Resources and Environmental Sciences. Chinese Academy of Sciences http://www.resdc.cn (accessed 2-10-2015).

(13) Chen, X.; Strokal, M.; Kroeze, C.; Ma, L.; Shen, Z.; Wu, J.; Chen, X.; Shi, X. Seasonality in river export of nitrogen: A modelling approach for the Yangtze River. *Science of the Total Environment* **2019**, *671*, 1282-1292.

(14) Liu, X.; Zhang, Y.; Han, W.; Tang, A.; Shen, J.; Cui, Z.; Vitousek, P.; Erisman, J. W.; Goulding, K.; Christie, P. Enhanced nitrogen deposition over China. *Nature* **2013**, *494* (7438), 459.

(15) Xu, W.; Luo, X.; Pan, Y.; Zhang, L.; Tang, A.; Shen, J.; Zhang, Y.; Li, K.; Wu, Q.; Yang, D. Quantifying atmospheric nitrogen deposition through a nationwide monitoring network across China. *Atmos. Chem. Phys.* **2015**, *15* (21), 12345-12360.

(16) Mayorga, E.; Seitzinger, S. P.; Harrison, J. A.; Dumont, E.; Beusen, A. H. W.; Bouwman, A. F.; Fekete, B. M.; Kroeze, C.; Van Drecht, G. Global nutrient export from WaterSheds 2 (NEWS 2): model development and implementation. *Environ. Model. Software* **2010**, *25* (7), 837-853.

(17) Bouwman, A. F.; Beusen, A. H. W.; Billen, G. Human alteration of the global nitrogen and phosphorus soil balances for the period 1970–2050. *Global. Biogeochem. Cy.* **2009**, *23* (4).

(18) Strahler, A. N. Quantitative analysis of watershed geomorphology. *Eos Transactions American Geophysical Union* **1957**, *38* (6).

(19) Döll, P.; Lehner, B. Validation of a new global 30-min drainage direction map. *J. Hydrol.* **2002**, *258* (1-4), 214-231.

(20) Beusen, A.; Van Beek, L.; Bouwman, A.; Mogollón, J.; Middelburg, J. Coupling global models for hydrology and nutrient loading to simulate nitrogen and phosphorus retention in surface water–description of IMAGE–GNM and analysis of performance. *Geosci. Model. Dev* **2015**, *8* (12), 4045.

(21) Leopold, L. B.; Wolman, M. G.; JP, M. Fluvial processes in geomorphology. *San Francisco, California, Freeman* **1964**, *522*, 135-163.

(22) Wollheim, W. M.; Peterson, B. J.; Thomas, S. M.; Hopkinson, C.; Vörösmarty, C. Dynamics of N removal over annual time periods in a suburban river network. *Journal of Geophysical Research: Biogeosciences* **2008**, *113* (G3).

(23) Liang, X.; Lettenmaier, D. P.; Wood, E. F.; Burges, S. J. A simple hydrologically based model of land surface water and energy fluxes for general circulation models. *J. Geophys. Res. Atmos.* **1994**, *99* (D7), 14415-14428.

(24) Chen, X.; Cui, Z.; Fan, M.; Vitousek, P.; Zhao, M.; Ma, W.; Wang, Z.; Zhang, W.; Yan, X.; Yang, J.; Deng, X.; Gao, Q.; Zhang, Q.; Guo, S.; Ren, J.; Li, S.; Ye, Y.; Wang, Z.; Huang, J.; Tang, Q.; Sun, Y.; Peng, X.; Zhang, J.; He, M.; Zhu, Y.; Xue, J.; Wang, G.; Wu, L.; An, N.; Wu, L.; Ma, L.; Zhang, W.; Zhang, F. Producing more grain with lower environmental costs. *Nature* **2014**, *514* (7523), 486-489. DOI: 10.1038/nature13609.

(25) Cui, Z. L.; Zhang, H. Y.; Chen, X. P.; Zhang, C. C.; Ma, W. Q.; Huang, C. D.; Zhang, W. F.; Mi, G. H.; Miao, Y. X.; Li, X. L.; Gao, Q.; Yang, J. C.; Wang, Z. H.; Ye, Y. L.; Guo, S. W.; Lu, J. W.; Huang, J. L.; Lv, S. H.; Sun, Y. X.; Liu, Y. Y.; Peng, X. L.; Ren, J.; Li, S. Q.; Deng, X. P.; Shi, X. J.; Zhang, Q.; Yang, Z. P.; Tang, L.; Wei, C. Z.; Jia, L. L.; Zhang, J. W.; He, M. R.; Tong, Y. A.; Tang, Q. Y.; Zhong, X. H.; Liu, Z. H.; Cao, N.; Kou, C. L.; Ying, H.; Yin, Y. L.; Jiao, X. Q.; Zhang, Q. S.; Fan, M. S.; Jiang, R. F.; Zhang, F. S.; Dou, Z. X. Pursuing sustainable productivity with millions of smallholder farmers. *Nature* **2018**, *555* (7696), 363-366. DOI: 10.1038/nature25785.

(26) Zhang, F.; Zhang, C. *High production and high efficiency of nutrient use management technology innovation and application*; China Agriculture University, 2017.

(27) Wang, X.; Dou, Z.; Shi, X.; Zou, C.; Liu, D.; Wang, Z.; Guan, X.; Sun, Y.; Wu, G.; Zhang, B.; Li, J.; Liang, B.; Tang, L.; Jiang, L.; Sun, Z.; Yang, J.; Si, D.; Zhao, H.; Liu, B.; Zhang, W.; Zhang, F.; Zhang, F.; Chen, X. Innovative management programme reduces environmental impacts in Chinese vegetable production. *Nature Food* **2021**, *2* (1), 47-53. DOI: 10.1038/s43016-020-00199-0.

(28) Data Center for Resources and Environmental Sciences Chinese Academy of Sciences Data Center for Resources and Environmental Sciences Chinese Academy of Sciences http://www.resdc.cn (accessed 2012).

(29) Yu, C.; Huang, X.; Chen, H.; Godfray, H. C. J.; Wright, J. S.; Hall, J. W.; Gong, P.; Ni, S.; Qiao, S.; Huang, G. Managing nitrogen to restore water quality in China. *Nature* **2019**, *567* (7749), 516.

(30) Ma, T.; Sun, S.; Fu, G.; Hall, J. W.; Ni, Y.; He, L.; Yi, J.; Zhao, N.; Du, Y.; Pei, T. Pollution exacerbates China's water scarcity and its regional inequality. *Nature Communications* **2020**, *11*.

(31) Schulte-Uebbing, L.; Beusen, A.; Bouwman, A.; de Vries, W. From planetary to regional boundaries for agricultural nitrogen pollution. *Nature* **2022**, *610* (7932), 507-512.

(32) Food and agriculture data. FAO. https://www.fao.org/faostat/en/#home (accessed.
(33) Bai, Z.; Ma, W.; Ma, L.; Velthof, G. L.; Wei, Z.; Havlík, P.; Oenema, O.; Lee, M. R.; Zhang, F. China’s livestock transition: Driving forces, impacts, and consequences. *Science advances* **2018**, *4* (7), eaar8534.
(34) MoA. Notice on implementation plan of ‘Zero-growth in Synthetic Fertilizer after 2020 Policy’. China, M. o. A. o. P. R., Ed.; Ministry of Agriculture of P.R. China.: 2015.
(35) SCPRC. *The Action Plan for Prevention and Control of Water Pollution*; State Council of the People’s Republic of China, 2015. http://www.gov.cn/flfg/2013-11/26/content_2535095.htm.
(36) SCPRC. the 14th Five-Year Plan of Green Agriculture Development. State Council of the People’s Republic of China: 2021.
(37) MEP. *National list of operating centralized wastewater treatment plants*; Ministry of Ecology and Environment of the People's Republic of China., 2014. http://www.mep.gov.cn/gkml/hbb/bgg/201506/W020150609575919731164.pdf.
(38) MEP. *National Intensive Monitoring and Control Enterprise List* Ministry of Ecology and Environment of the People's Republic of China., 2016. http://www.mep.gov.cn/gkml/hbb/bgt/201602/t20160204_329897.htmDOI: 000014672.
(39) National Surface Water Database. Shanghai Qingyue. http://data.epmap.org/water (accessed 2020/10/11).
(40) Map of Surface Water Quality. . Institute of Public and Environmental Affairs. http://www.ipe.org.cn/MapWater/water.html?q=2 (accessed 2020/10/11).
